# Polyacrylamide hydrogel-immobilized *Escherichia coli* cell lysate for efficient removal and reduction in transformability of extracellular antibiotic resistance genes in water

**DOI:** 10.1128/aem.00253-26

**Published:** 2026-05-06

**Authors:** Hua Li, Fangjuan Li, Yajie Shi, Xinxin Wang, Xiaomeng Wang, Guanyu Zheng, Lixiang Zhou, Barth F. Smets

**Affiliations:** 1 Department of Environmental Engineering, College of Resources and Environmental Sciences, Nanjing Agricultural University 98429, Nanjing, China; 2 Jiangsu Collaborative Innovation Center for Solid Organic Waste Resource Utilization, Nanjing, China; 3 Department of Biological and Chemical Engineering-Environmental Engineering, Water Center for Water Technology, Aarhus University 1006, Aarhus, Denmark; University of Minnesota Twin Cities, St. Paul, Minnesota, USA

**Keywords:** extracellular antibiotic resistance genes, transformation, cell lysate, cell immobilization

## Abstract

The dissemination of extracellular antibiotic resistance genes (eARGs) through horizontal gene transfer poses a serious threat to global water safety. While *Escherichia coli* MG1655 is known to employ intracellular nucleases to defend against foreign DNA, the potential of its cell lysate to enzymatically degrade plasmid-borne eARGs and disrupt their environmental propagation remains unexplored. This study demonstrated that *E. coli* MG1655 cell lysate can degrade the plasmid-borne *amp^R^* gene by 99.9% within 48 h, and the maximum first-order degradation rate constant measured by long amplicon qPCR was 0.20 h^−1^ at the selected dosage state. This degradation eliminated the ability of the plasmid pUC19 for transformation, reducing it to below detectable levels (<10^−6^ transformants/recipients). The DNase I, produced by the chromosomal *endA* gene, was identified as the likely nuclease driving eARG cleavage. Environmental conditions critically modulated degradation efficiency: humic acid (50 mg/L) decreased the rate by 41.3%, while divalent cations (e.g., Mg^2+^) increased the rate by 1.65-fold. To transmit laboratory findings into real-world application, a polyacrylamide hydrogel-immobilized lysate (PAM-cell lysate) was engineered, which outperformed free lysate in treating plasmid-spiked real wastewater, sustaining 99.9% of *amp^R^* removal across three treatment cycles. This research demonstrated that PAM-cell lysate, without genetic modification, can be a cost-effective and scalable solution for attenuating the spread of eARGs in water, advancing the development of complementary enzymatic strategies for sustainable water remediation.

**IMPORTANCE:**

The mitigation of extracellular antibiotic resistance genes (eARGs) is an urgent priority for controlling the spread of antibiotic resistance via natural transformation. Developing environmentally benign strategies to effectively degrade plasmid-borne eARGs and prevent their transformation is therefore essential. However, few studies have explored the potential of bacterial cell lysates retaining native nuclease activity for eARG removal. In this work, we demonstrate that a candidate *Escherichia coli* cell lysate can rapidly degrade the plasmid pUC19 carrying the *amp^R^* gene, with DNase I identified as the primary degradative enzyme. Furthermore, we show that immobilized *E. coli* cell lysate can concurrently adsorb and enzymatically degrade eARGs, effectively suppressing horizontal gene transfer via transformation. These findings highlight a novel, cost-effective, and scalable biocatalytic strategy for controlling the dissemination of eARGs in water treatment and environmental systems.

## INTRODUCTION

Antibiotic resistance is a grave global public health concern (1–3). Horizontal gene transfer, facilitated by mobile genetic elements like plasmids, plays a significant role in the rapid spread of antibiotic resistance genes (ARGs) in the aquatic environment (3, 4). Transformation, where competent cells directly take up extracellular ARGs (eARGs), is an important pathway of horizontal gene transfer. Several studies have shown that a ratio of approximately 2.0 × 10^−4^ competent cells to all bacterial cells exists in the aquatic environment (5–7). eARGs can persist in the environment for months or even years, emphasizing the importance of developing effective methods for their removal (4, 8, 9). Therefore, the removal of eARGs is urgent and crucial for controlling eARG-mediated transformation.

eARGs are susceptible to damage through physical and chemical processes. However, traditional disinfection technologies, particularly chlorination at non-lethal concentrations, have been found to enhance the transformative uptake of plasmid-borne eARG by increasing intracellular oxidative stress and membrane permeability of the recipient cells (4, 10). To address this challenge, it is crucial to develop safe, environmentally friendly, and cell-free enzymatic treatment strategies that effectively degrade plasmid-borne eARGs and prevent their transformation without generating hazardous byproducts.

Given these limitations, enzymatic approaches targeting eARGs offer a promising alternative due to their specificity and low environmental impact. The bacterium *Vibrio cholerae* has been reported to produce extracellular nuclease, Dns, to significantly decrease its transformability by degrading extracellular DNA (11). Furthermore, the nuclease-producing bacterium *Deinococcus radiodurans* has been found to synthesize extracellular nucleases to degrade *tetA*, *aphA*, and *blaTEM-2* carried by plasmid RP4, thereby mitigating the transformation of these eARGs into *Enterococcus faecalis* (12). Additionally, it was reported that diverse bacteria including *Aeromonas*, *Streptomyces*, *Bdellovibrio bacteriovorus* HD, *Shewanella*, *Staphylococcus*, *Neisseria*, and *Haemophilus,* secrete nucleases capable of degrading extracellular DNA (12–14). These findings have sparked interest in utilizing the nuclease-producing bacteria for the removal of eARGs in water (14, 15). However, while this approach has low ecotoxicity and avoids the production of harmful by-products (12, 16), the introduction of the nuclease-producing bacteria, such as *D. radiodurans* and *S. oneidensis*-based engineered DNA scavenger, may face the limitations of potential biosafety risks and poor survival or washout of the introduced strains under dynamic wastewater conditions (15). Therefore, developing cell-free enzymatic systems that retain nuclease activity without relying on live bacteria represents an opportunity for sustainable eARG mitigation.

*Escherichia coli* MG1655, an important host for metabolic engineering, has been widely utilized for the production of value-added chemicals (17). Notably, approximately 50 nucleases have been identified in *E. coli* (18). A well-known pathway for DNA fragmentation is DNase-mediated DNA hydrolysis, which results in deoxyribose, orthophosphate, and deoxynucleosides (12, 19, 20). Specifically, DNase I has been reported to cleave the phosphodiester backbone of the extracellular DNA double helix in the presence of divalent cations, and it introduces single-stranded nicks by hydrolysis of the P-O_3_′-bond and produces 5′-phosphorylated fragments (21). Moreover, *E. coli* MG1655 exhibits low competency due to its robust defense system against exogenous DNA (17). This defense system primarily involves the DNA-specific endonuclease I *EndA*, which aids in the production of DNase I capable of degrading foreign double-stranded DNA (17, 22). Additionally, the presence of the *recA* gene in the *E. coli* MG1655 genome also contributes to the instability of plasmid DNA (17). We infer that cell lysates of *E. coli* MG1655 contain DNase, making it theoretically feasible to utilize these lysates for the degradation of eARG.

Free nucleases often suffer from low stability, activity, and concentration, along with limited reusability in complex environments (20, 23). Moreover, various ions can affect the enzymatic function of nucleases either as cofactors or as inhibitors (24). Immobilization techniques have been developed to address these issues by enhancing nuclease stability, improving access to target DNA, increasing enzyme concentration, and enabling convenient nuclease reuse in wastewater treatment processes (25). One common approach of enzyme immobilization is through entrapment within a host matrix, with polyacrylamide (PAM) being a popular choice due to its favorable mechanical stability, environmental friendliness, high biocompatibility, and cost-effectiveness (26). Therefore, this study aims to investigate the potential of polyacrylamide hydrogel-immobilized lysate (PAM-cell lysate) in enhancing the removal of plasmid-borne eARGs during a continuous cyclic degradation process. We hypothesize that *E. coli* MG1655 cell lysate can degrade plasmid-borne eARGs and that immobilization in PAM enhances its stability for wastewater treatment applications.

The primary objectives of this study were 2-fold: (i) to thoroughly assess the ability of *E. coli* MG1655 cell lysate to degrade plasmid-borne eARGs in comparison to lysate from relevant environmental bacteria (*Acinetobacter baylyi* ADP1, *Pseudomonas putida* KT2440, and *Acidithiobacillus ferrooxidans* LX5) and (ii) to elucidate the mechanisms behind this process and its applicability in the environment. Specifically, we examined the degradation kinetics of the plasmid pUC19 carrying the *ampR* gene and its subsequent loss of transformability, while investigating if this process is consistent across different *E. coli* subspecies (JM109, BL21(DE3), and HB101). To unravel the degradation mechanisms, we performed strand break analysis, deoxynucleoside damage quantification, and EPS interference assays, alongside profiling DNase I activity and conducting inhibitor experiments. Furthermore, we evaluated how key wastewater matrix components (i.e., humic acid and divalent cations) influence degradation efficiency. Finally, we engineered a PAM-immobilized cell lysate (PAM-cell lysate) and compared its performance to free lysate in treating real wastewater under continuous cycles. Overall, this study demonstrates the potential of bacterial cell lysate as a powerful and scalable catalyst for reducing the spread of eARGs in aquatic environments and provides valuable insights for future nuclease-based water treatment strategies.

## MATERIALS AND METHODS

### Plasmid, enzymes, chemicals, competent cells, and strains

The pUC19 plasmid (500 ng/μL) (Sangon Biotech Co., China), carrying the *amp^R^* as a model eARG (Fig. S1), was utilized in this study. Enzymes, including deoxyribonuclease I (DNase I), alkaline phosphatase (ALPase), and phosphodiesterase I, were also purchased from Sangon Biotech Co., Ltd. in China. The deoxyribonucleotides, namely deoxyguanosine (dG), deoxyadenosine (dA), deoxythymidine (dT), and deoxycytidine (dC), were purchased from Sigma-Aldrich. Trelief 5α chemically competent cells, a mutant of *E. coli* DH5α, were obtained from Tsingke Biotechnology Co., Ltd. The bicinchoninic acid (BCA) protein assay kit was purchased from Suzhou Keming Biotechnology Co., Ltd. The SanPrep Column PCR Product Purification Kit was obtained from Sangon Biotech, Shanghai, China. Four bacteria (*E. coli* MG1655, *A. baylyi* ADP1, *P. putida* KT2440, and *Acidithiobacillus ferrooxidans* LX5) in our lab were selected as potential candidates for the preparation of the cell lysate. Additionally, *E. coli* JM109, *E. coli* BL21(DE3), and *E. coli* HB101, in comparison to *E. coli* MG1655, were purchased from Shanghai Weidi Biotechnology Co., Ltd. Details of bacterial culture methods are described in Text S1. The broad-spectrum nuclease inhibitor, a mixture of recombinant protein and Gentamicin sulfate, was obtained from Shanghai Beibo Biology Co., Ltd. The DNase-I ELISA Kit was obtained from Shanghai Enzyme-linked Biotechnology Co., Ltd. Acrylamide and N,N′-methylene-bis-acrylamide (MBA), used as a cross-linker for cell lysate immobilization, were purchased from Aladdin Chemistry Co., Ltd. in Shanghai.

### Preparation of bacterial cell lysate

Four candidate bacterial strains, including *E. coli* MG1655, *A. baylyi* ADP1, *P. putida* KT2440, and *A. ferrooxidans* LX5, which are widely distributed in the natural environment, were selected for the preparation of bacterial cell lysates. Additionally, three subspecies of *E. coli*, namely *E. coli* JM109, *E. coli* BL21(DE3), and *E. coli* HB101, were utilized for comparison with *E. coli* MG1655. The robustness of *E. coli* MG1655 was assessed in comparison to its sibling strains using a previously established method described in Text S2 (17). The detailed process for preparing the bacterial cell lysate is illustrated in Fig. S2. Initially, the bacterial cells were counted using a microscope after culturing, with an initial cell concentration of approximately 1 × 10^9^ cells/mL. The cells were then centrifuged at 8,000 × *g* for 10 min, followed by a washing step to remove impurities. Subsequently, the cells were resuspended in Tris-HCl (pH 7.1, 10 mM). Twenty milliliters of the resuspended bacterial cell samples were homogenized and disrupted by ultrasonication on ice. The optimized ultrasonication process was conducted on ice to dissipate heat, at a power density of 135 W/mL for 7 min, with intervals of 10 s on and 3.3 off to prevent overheating and protein denaturation. Afterward, the samples were centrifuged at 8,000 rpm for 10 min at 4℃. The resulting supernatant was filtered through a 0.22-μm syringe filter to obtain the crude cell lysate for the following experiment. The efficiency of cell fragmentation was determined by plate counting and calculated according to equation (1). Furthermore, a heat-inactivated cell lysate (treated for 30 min at 95°C) was used as a negative control for comparison.


(1) $$\text{ Cell fragmentation efficiency }=1-\frac{\text{ number of bacterial cells after cell fragmentation }}{\text{ number of original bacterial cells }}$$

### Plasmid degradation and the effects of environmental factors on eARG degradation by *E. coli* MG1655 cell lysate

To assess plasmid degradation, 495 μL of each bacterial cell lysate (in Tris-HCl, pH 7.1, 10 mM) was mixed with 5 μL of plasmid pUC19 (10 ng/μL), resulting in a final concentration of 0.1 ng/μL. This concentration is environmentally relevant and commonly employed in plasmid degradation studies. It was specifically utilized to simulate a worst-case pollution scenario and provide sufficient material for the subsequent transformation assays (27–30). The mixture was then incubated in the dark at 37°C for a continuous period of 48 h. The concentration of the extracted cell lysate from the selected bacteria was determined using a bicinchoninic acid (BCA) protein assay kit, as shown in Table S1. To eliminate the impact of differences in cell lysate concentration among the four candidate bacterial cell lysates on eARG degradation, changes in *amp^R^* gene copy number were compared per unit lysate concentration (expressed as the protein concentration) among the four candidate bacterial cell lysates. The ratio of cell lysate to sterile water was set at 0:1, 1:4, 3:2, and 1:0, resulting in final protein concentrations of 0, 0.08, 0.24, and 0.4 mg/mL, respectively. These concentrations are equivalent to the lysis of 0, 2 × 10^8^, 6 × 10^8^, and 1 × 10^9^ cells/mL of *E. coli* MG1655 cells. The impacts of the concentrations of *E. coli* MG1655 cell lysate (0.08, 0.24, and 0.40 mg/mL) on the degradation of eARG (0.1 ng/μL) were investigated. Both heat-inactivated (30 min at 95°C) cell lysate and nuclease-free water were included as negative and blank controls, respectively. At intervals of 0, 24, 36, and 48 h, the reaction solution was vortexed, and 100 μL of each solution was extracted to determine the *amp^R^* copy number using both short and long amplicon qPCR as the method described in “Plasmid purification and quantitative real-time PCR (qPCR),” below.

As natural organic matter is known to protect eARG from degradation by DNase through adsorption, we investigated the impact of humic acid (HA) at environmentally relevant concentrations of 5 and 50 mg/L on the degradation of eARG by cell lysate (31). In addition, the influence of coexisting metal ions at environmentally relevant concentrations (32, 33), namely Ca^2+^(1 mM), Mg^2+^(1 mM), Fe^3+^(0.05 mM), and Al^3+^(0.05 mM), on *amp^R^* removal was assessed in the presence of cell lysate (0.40 mg/mL). After 48 h, the samples were taken from the reaction solution to determine *amp^R^* copy number using a short amplicon analysis.

### eARG transformation experiments

*E. coli* TreliefTM5α was used as the competent cell to assess the transformation potential of plasmid-borne eARGs post-treatment with the cell lysate of *E. coli* MG1655. Briefly, the pUC19 plasmid (10 ng/μL) was subjected to *E. coli* MG1655 cell lysate treatment (0, 0.08, 0.24, and 0.4 mg/mL) for 48 h, and then, the plasmid was purified using a PCR Product Purification Kit. Subsequently, 50 μL of thawed competent *E. coli* TreliefTM5α cells were mixed with 100 μL of purified plasmid. The resulting mixture was incubated on ice for 30 min, followed by a heat shock at 42°C for 45 s. The sample was then returned to ice for an additional 2 min, and 250 μL of sterile LB medium was added into the bacterial solution afterward. The bacterial solution was cultured at 37°C for 1 h with agitation at 150 rpm. After serial dilution of the original cell suspension, 100 μL of each dilution was plated onto selective LB agar plates containing 100 mg/L ampicillin, as well as antibiotic-free plates, to determine the CFU number (34). The colonies on the agar plate were counted after incubation at 30°C for 24 h. The efficiency of transformation was calculated using equation (2), where the transformant colony-forming unit (CFU) and total CFU represented the colony forming on LB with and without ampicillin, respectively.


(2) $$\text{ Transformation efficiency }=\frac{\text{ transformant CFU }}{\text{ total CFU }}$$

### Plasmid purification and quantitative real-time PCR (qPCR)

Following incubation with cell lysate, the pUC19 plasmid was purified using a SanPrep Column PCR product purification kit (Sango Biotech, Co., China) to remove protein and salt contaminants. A short amplicon qPCR specifically targeted a 178 bp region of the *amp^R^* gene, while a long-amplicon qPCR covered the entire 861 bp length of *amp^R^*, providing a comprehensive assessment of *amp^R^* integrity (35). The short-amplicon qPCR exhibited higher amplification efficiency and reduced nonspecific amplification, although it may underestimate the extent of plasmid damage (36). The sequences of primers were designed using the Primer-Blast in the National Center for Biotechnology Information (NCBI) and are provided in Table S2. The detailed qPCR reaction system and operating procedures are shown in Text S3. The standard curves for the short and long amplicons of the *amp^R^* gene are shown in Table S3.

### Measurement and gel electrophoresis analysis of plasmid pUC19 damage

Bases damage and strand breaks are two major forms of DNA lesions (37). In this study, damage to plasmid pUC19 was measured following a well-established protocol (28). Specifically, 1.4 μg of plasmid pUC19 treated with varying concentrations of *E. coli* MG1655 cell lysate were purified using a PCR product purification kit to assess damage to the plasmid. The purified plasmids were then subjected to total hydrolysis using 1.0 U of DNase I, 0.1 U of phosphodiesterase I, and 1.0 U ALPase in a Tris-HCl buffer (pH = 7.6) at 37°C for 24 h to determine the amount of pUC19 remaining after treatment with cell lysate by measuring the residual concentration of deoxynucleosides post the hydrolysis process. To avoid interference from deoxyguanosine oxidation during the hydrolysis process, 1 mM of deferoxamine mesylate was included in the reaction solution. Following hydrolysis, the concentration of each deoxynucleoside was quantified utilizing high-performance liquid chromatography (HPLC). The chromatographic parameters and the standard curves of four deoxynucleosides (dG, dA, dT, and dC) are shown in Text S4 and Table S4, respectively.

Gel electrophoresis was performed to assess strand breaks in the pUC19 plasmid (initial concentration of 10 ng/μL) before and after treatment with varying concentrations of *E. coli* MG1655 cell lysate, as described in “Plasmid degradation and the effects of environmental factors on eARG degradation by *E. coli* MG1655 cell lysate,” above. Agarose gel electrophoresis was conducted as per a previous method (28). Linearized pUC19 was obtained by incubating the plasmid with the type II restriction enzyme QuickCut *EcoR I* at 37°C for 1 h, followed by enzyme inactivation at 65°C for 20 min. The detailed experimental procedure for gel electrophoresis is shown in Text S5. Furthermore, the impact of *E. coli* MG1655 cell lysate on the degradation of plasmid RSF1010 with streptomycin resistance and the pCLT cloning vector with kanamycin resistance was assessed through gel electrophoresis analysis.

### Determination of the existence and the activity of DNase I

To exclude the influence of bacterial extracellular polymeric substance (EPS) on the degradation of pUC19, EPS was prepared from *E. coli* MG1655 cells, and EPS-depleted *E. coli* MG1655 cell lysates were prepared according to the procedure outlined in Text S6. Cell lysates from both EPS-depleted *E. coli* MG1655 and *E. coli* MG1655 cells were prepared as detailed in “Preparation of bacterial cell lysate,” above. The effects of soluble EPS, EPS-depleted cells, and cell lysate on the degradation of pUC19 (0.1 ng/μL) were assessed using the method described in “Plasmid degradation and the effects of environmental factors on eARG degradation by *E. coli* MG1655 cell lysate,” above. The copies of eARG were quantified within 48 h using a short amplicon analysis. The presence of *recA* and *endA* genes in all bacterial strains utilized in our study was confirmed through colony PCR, with detailed methodology and primer information provided in Text S7 and Table S5, respectively. Ethylenediaminetetraacetic acid (EDTA) is a well-known metal ion chelator that inhibits enzymes requiring divalent metal ions, such as DNase I (32, 38). EDTA (10 and 20 mM) was individually added to cell lysate solutions containing 10 ng/μL pUC19 to observe the inhibition effect. Various concentrations of nuclease inhibitor (0.5, 1, and 2 mM) were also used to inhibit the nuclease activity. The mixtures were incubated at 37°C and sampled after 48 h. The inhibition effects were immediately analyzed using agarose gel electrophoresis. DNase I activity was measured using the DNase-I ELISA Kit according to the manufacturer’s instructions (39), and the standard curve of DNase I is shown in Fig. S3. Homologous sequences of the *End A* protein (NCBI Reference Sequence: NC_000913.3) were retrieved by conducting a BLAST search against the non-redundant protein sequence (nr) database at the NCBI. Multiple sequence alignment was performed using the MUSCLE algorithm in Mega-X software (40), followed by trimming with trimAl (41). After removing sequences exhibiting 100% identity, a phylogenetic tree was constructed in MEGA-X via the neighbor-joining method with 1,000 bootstrap replicates.

### The immobilization of bacterial cell lysates by PAM hydrogel and its impact on the degradation of pUC19 plasmid in water

The PAM-entrapped *E. coli* MG1655 cell lysate was prepared following the procedures outlined in a previous study (26), with detailed information provided in Text S8. Twenty milliliters of *E. coli* MG1655 cell lysate were used to prepare the PAM-cell lysate. The morphology of the PAM-cell lysate was studied using a scanning electron microscope (SEM) (Hitachi Regulus8100, Japan). Nitrogen adsorption-desorption analysis was performed on the PAM-cell lysate using the Micromeritics 3Flex Surface Characterization Analyzer (Micromeritics 3Flex). The surface area and pore size distribution were calculated using the Brunauer-Emmett-Teller (BET) model. The droplet contact angle of the PAM-cell lysate was measured with an EasyDrop goniometer (Attension Theta Flex, Biolin Scientific, Sweden) (42). The swelling characteristics were tested according to a previous study (26), and the detailed procedures are shown in Text S9.

To investigate the degradation effects of PAM-cell lysate on eARG, the efficiency of eARG degradation was compared among free cell lysate, PAM-cell lysate, and heat-deactivated PAM-cell lysate treatments in pure water. A total of 250 mg of PAM-cell lysate was added to 50 mL of sterilized ultrapure water, corresponding to a protein concentration of 0.4 mg/mL (80 mg protein/g PAM-cell lysate), along with a concentration of 0.1 ng/μL of pUC19. These mixtures were then subjected to magnetic stirring at 37°C and 180 rpm for a duration of 48 h. Equal amounts of free cell lysate and heat-deactivated PAM-cell lysate samples were also included as controls. The concentration of *amp^R^* was determined within 48 h.

To assess the efficiency of eARG removal by PAM-cell lysate in real wastewater, a cyclic degradation experiment was conducted using wastewater collected from the effluent in the Taihu New City Wastewater Treatment Plant (WWTP) in Wuxi. The wastewater was filtered through a 0.22-μm filter membrane before use. The composition characteristics of the wastewater were as follows: pH 6.9, TOC 45.6 mg/L, NH_3_-N 0.2 mg/L, and COD 57.7 mg/L. In the experiment, 150 mg of PAM-cell lysate was added to 30 mL of wastewater, with the initial concentration of the pUC19 plasmid set at an environmentally relevant level of 10⁵ copies/μL. A negative control was established by adding deactivated PAM-cell lysate. The mixtures were magnetically stirred at 37°C, and each cycle lasted for 24 h. After each cycle, the liquid supernatant was removed through static settlement, and 30 mL of the wastewater containing the same concentration of pUC19 was used for the next cycle. eARG was extracted from the wastewater, and a short amplicon was used to determine the *amp^R^* concentration. Furthermore, the TOC concentration was measured after each cycle using a TOC analyzer (Shimadzu, TOC-L, Japan) to assess any changes in TOC concentration during treatment with PAM-cell lysate.

### Data treatment and statistics

Figures were generated using Origin 2016 software (OriginLab, USA). Statistical analysis was conducted using SPSS software version 17.0 (Armonk, NY, USA). Significant differences were identified through a one-way ANOVA with Student Newman-Keuls (SNK) post-hoc test. Differences were considered significant at a *P* value less than 0.05.

## RESULTS

### Degradation of eARGs by *E. coli* MG1655 cell lysate

**Fig 1:**
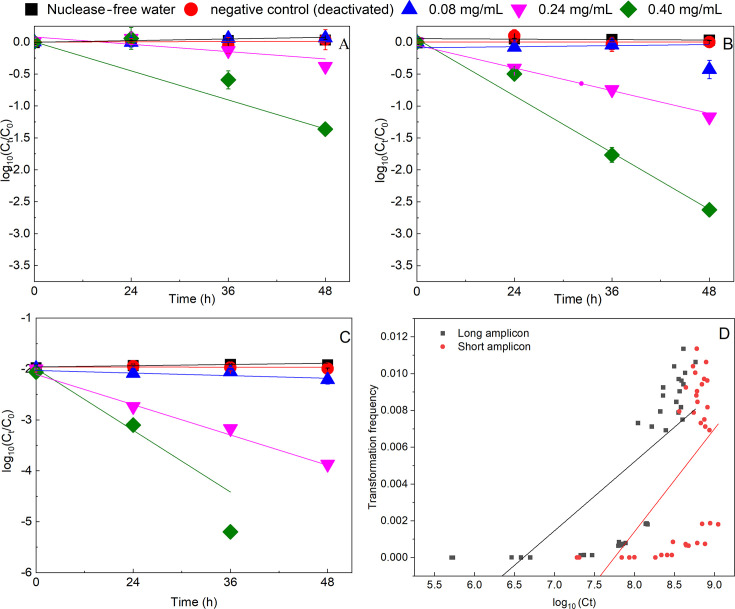
Changes in the concentration and transformation frequency of the *amp^R^* gene treated with different concentrations of cell lysate from *E. coli* MG1655 over 48 h. (**A**) *amp^R^* gene copy determined by short amplicon PCR (178 bp); (**B**) *amp^R^* gene copy determined by long amplicon PCR (861 bp); (**C**) transformation frequency determined by plate counting; and (**D**) correlations between transformation frequency and copy of *amp^R^* gene determined by short amplicon and long amplicon PCR. The symbols represent the measured data, and the error bars represent the standard deviation from the triplicate experiment. Lines are the linear regressions of the experimental data.

The cell fragmentation efficiency of *E. coli* MG1655 was 97.1%, suggesting an effective lysis of the bacterial cells. The cell lysate of *E. coli* MG1655 exhibited the maximum reduction of eARGs, reaching a 1.1 log reduction per unit protein concentration of cell lysate at 48 h (Fig. S4) (*P* < 0.05). In contrast, the cell lysates of *A. ferrooxidans LX5*, *A. baylyi ADP1*, and *P. pudita KT2440* did not show significant impacts on eARG degradation over time (*P* > 0.05) (Fig. S4). The results of the short amplicon qPCR analysis revealed that high concentrations of *E. coli* MG1655 cell lysate (0.24 and 0.40 mg/L) effectively removed approximately 0.38 log and 1.36 log of the *amp^R^* gene within 48 h (*P* < 0.05) (Fig. 1A). However, no significant reduction was observed when using a lower concentration of cell lysate (0.08 mg/L) (*P* > 0.05) (Fig. 1A). The rapid removal stage, which occurred after 24 h, followed a first-order kinetic model (Fig. 1). The degradation rate constant (k) values determined from the slope of the linear plot (k = In (10) × slope) were 0.055 (*R^2^* = 0.994) and 0.12 h ^−1^(*R^2^* = 0.999) for the 0.24 and 0.40 mg/mL cell lysate treatment, respectively (Table 1). Moreover, the long amplicon qPCR analysis yielded higher rate constants, with respective k values of 0.074 (*R^2^* = 0.962) and 0.20 h ^−1^(*R^2^* = 0.999) for the 0.24 mg/mL and 0.40 mg/mL cell lysate treatments, respectively (Table 1). No significant reduction in eARG copies was observed in the negative control, nuclease-free water, and heat-inactivated cell lysate.

**TABLE 1:** First-order rate constants of eARG degradation measured by short-amplicon qPCR (SA-qPCR) and long-amplicon qPCR (LA-qPCR), and the eARG transformation frequencies

	Result at cell lysate dosage (mg/mL) of:		
	0.08	0.24	0.4
eARG degradation rate (SA-qPCR) (h^−1^)	0.0035 ± 0.00097	−0.055 ± 0.011	−0.12 ± 0.026
eARG degradation rate (LA-qPCR) (h^−1^)	−0.016 ± 0.0044	−0.074 ± 0.0065	−0.20 ± 0.0030
Transformation frequency (h^−1^)	−0.0091 ± 0.00063	−0.087 ± 0.0040	−0.19 ± 0.0040

### Reduction of eARG transformability by *E. coli* MG1655 cell lysate

The corresponding decay rate constant for transformation potential increased from 0.0091 to 0.19 h^−1^ as the concentration of cell lysate increased from 0.08 mg/L to 0.4 mg/L, indicating that higher concentrations of cell lysate could accelerate the decline in transformation frequency (Fig. 1C; Table 1). After 48 h of treatment with 0.4 mg/L of cell lysate, eARG could still be detected by qPCR (Fig. 1A and B), while the transformation frequency dropped significantly and dropped below the detection limit (approximately 10^−6^ T/R) (Fig. 1C). These results indicate that eARGs degraded by the cell lysate are no longer taken up by the recipient bacteria. Moreover, there is a significant correlation between the transformation frequency and the abundance of the *amp^R^* gene determined by short amplicon (*r* = 0.56, *P* < 0.001) and long amplicon (*r* = 0.77, *P* < 0.001), respectively (Fig. 1D).

### Structure changes in the pUC19 plasmid following exposure to the *E. coli* MG1655 cell lysate

**Fig 2:**
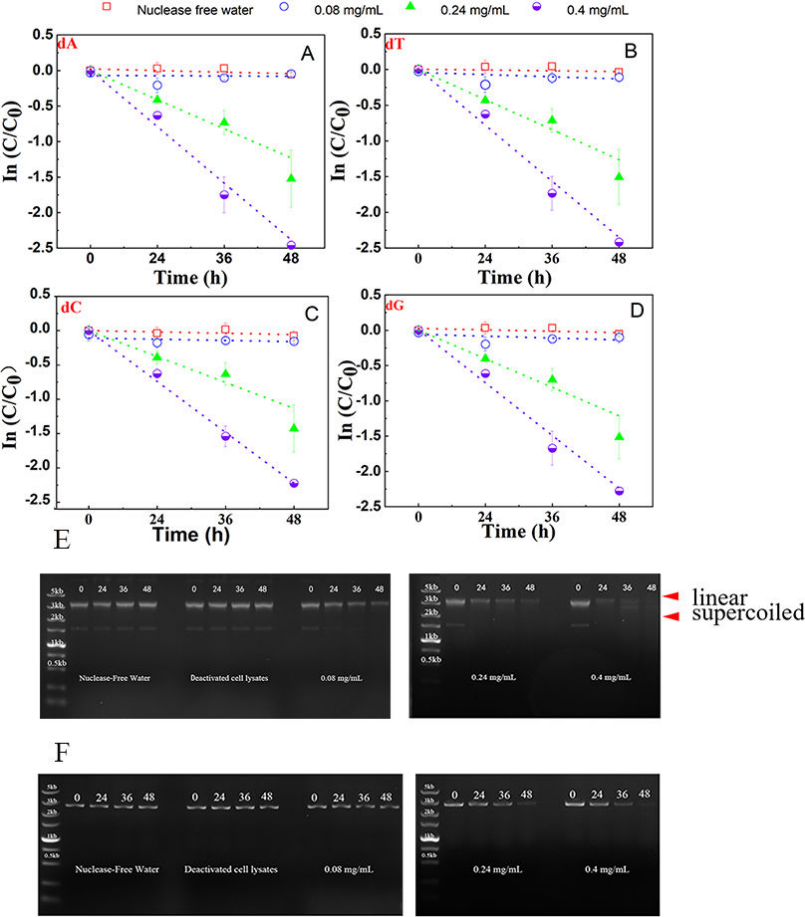
Changes in the natural logarithm of the post-digested remaining concentration of deoxynucleoside in the residual plasmid treated with different concentrations of *E. coli* MG1655 cell lysate (0.08, 0.24, and 0.4 mg/L) for 48 h. (**A**) dA; (**B**) dT; (**C**) dC; (**D**) dG. (**E**) Gel electrophoresis of pUC19 plasmid treated with nuclease-free water, deactivated cell lysates, and different concentrations of cell lysates (0.08, 0.24, and 0.4 mg/L) at different time points. (**F**) Gel electrophoresis of pUC19 plasmid treated with different concentrations of cell lysates (0.08, 0.24, and 0.4 mg/L) at different time points, followed by digestion with the restriction enzyme *EcoRI*. Lines are the linear regressions of the experimental data.

Loss of the four deoxynucleosides was found to increase with higher concentrations of cell lysate, in accordance with a first-order kinetic model (Fig. 2A through D). The removal efficiencies and first-order rate constants for base damages of the four deoxynucleosides were uniform (Tables S6 and S7), and the degradation rate constant k was positively correlated with the concentration of cell lysate (*r* = 0.968, *P* < 0.05), suggesting that there was no preferential removal of the four deoxynucleosides by *E. coli* MG1655 cell lysate. Gel electrophoresis experiments were performed to explore plasmid strand breaks. In Fig. 2E and F, the intact pUC19 band generally exists in a supercoiled form, which is located lower than the pUC19 linearized by *EcoRI* (34, 35). The negative controls without cell lysate and those with heat-inactivated cell lysate showed no significant degradation of eARG, as indicated by the clear agarose gel electrophoresis bands (Fig. 2E and F), indicating the thermal instability of *E. coli* MG1655 cell lysate. Following treatment of the DNA with cell lysate, we observed complete disappearance of the supercoiled band and increased smearing in linear DNA bands (Fig. 2E). This effect was most notably observed at a lysate concentration of 0.24 mg/mL following 36 h of incubation (Fig. 2E). These findings suggest that double-strand breaks drive the conformational transition from supercoiled to linear DNA, followed by progressive fragmentation of linear plasmids during prolonged lysate exposure.

### Existence of DNase in *E. coli* MG1655 cell lysate

**Fig 3:**
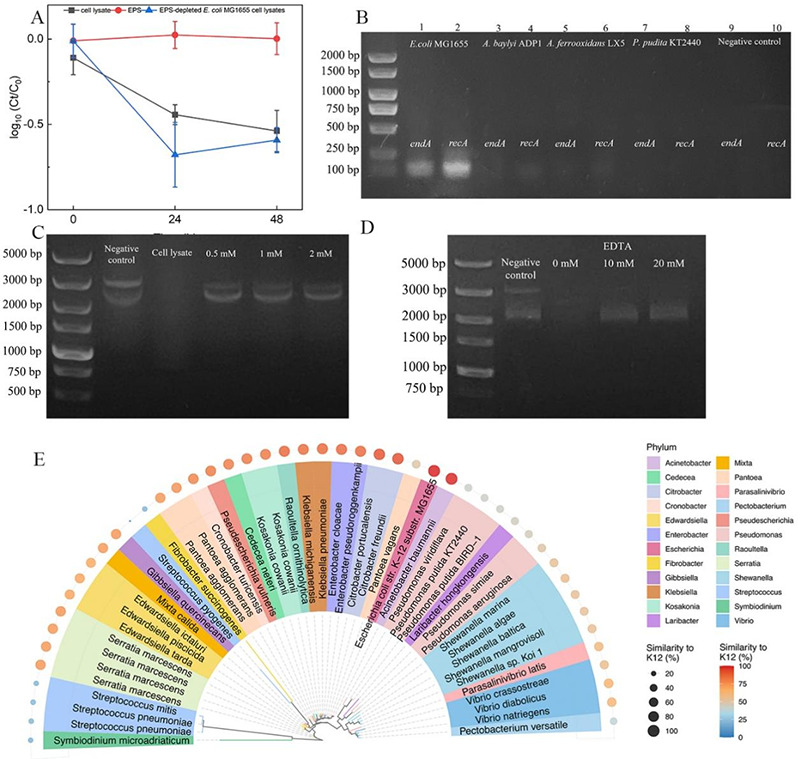
Evidence of eARG degradation by existing DNase in the cell lysate of *E. coli* MG1655. (**A**) Changes in the logarithm of eARG copies degraded by the cell lysate, EPS fraction, and EPS-depleted *E. coli* MG1655 cell lysates. Cell lysate, soluble EPS, and EPS-depleted *E. coli* MG1655 cell lysates were prepared by ultrasonic splitting of 10^9^ cells/mL of *E. coli* MG1655 (WT). (**B**) Identification of the *endA* and *recA* genes in *E. coli* MG1655, *A. baylyi* ADP1, *A. ferrooxidans* LX5, and *P. putida* by agarose gel electrophoresis. (**C**) Gel electrophoresis of plasmid pUC19 after 48 h of co-incubation with cell lysate or cell lysate spiked with different concentrations of a nuclease inhibitor. (band 1: negative control; band 2: co-incubation with cell lysate (0.24 mg/L); band 3–5: co-incubation with cell lysate (0.24 mg/L) spiked with different concentrations of nuclease inhibitor (0.5, 1, and 2 mM). (**D**) Gel electrophoresis of plasmid pUC19 after 48 h of co-incubation with cell lysate (0.24 mg/mL) or the cell lysate spiked with different concentrations of EDTA (10 mM and 20 mM) (band 1: negative control; band 2: co-incubation with cell lysate; band 3–4: co-incubation with cell lysate spiked with different concentrations of EDTA (10 mM and 20 mM) to assess the degradation of plasmid pUC19. (**E**) The distribution of protein *endA* in the selected bacteria (shown in phylogenetic tree). Matching scores reflect the amino acid sequence identity of the EndA protein, similarity between *E. coli* MG1655 and other bacteria. The larger circular legend represents greater similarity.

In the presence of *E. coli* MG1655 cell lysate, eARG levels continuously decreased over time, and there was no significant difference in the log reduction values between the cell lysate and the EPS-depleted cell lysate (*P* > 0.05) (Fig. 3A). In contrast, no significant degradation of eARG was observed in the EPS solution over the course of 48 h (Fig. 3A). These results collectively demonstrate that the EPS alone is incapable of degrading eARGs, and the degradation capacity of the cell lysate likely relies on the presence of intracellular nuclease.

Agarose gel electrophoresis exhibited the presence of *endA* and *recA* genes in the genome of *E. coli* MG1655, while the other candidate bacteria lack these genes (Fig. 3B). Furthermore, the concentration of DNase I in cell lysates of four *E. coli* strains was significantly higher compared to the other candidate bacteria (Fig. S5) (*P* < 0.05). Notably, the cell lysate of *E. coli* MG1655 demonstrated its ability to nonspecifically degrade double-stranded DNA, such as pUC19, RSF1010, and pCLT (Fig. S6).

We used a DNase inhibitor to investigate whether DNase was involved in eARG degradation. Gel electrophoresis experiments revealed that the DNase inhibitor effectively inhibited the degradation of eARG-carrying pUC19 plasmid even at a concentration of 0.5 mM (Fig. 3C). Additionally, the addition of EDTA (10 mM and 20 mM) resulted in the inhibition of plasmid degradation by the cell lysate (Fig. 3D).

Since the presence and sequence homology of *endA* in bacterial strains may indicate their potential for DNase production via cell lysis, we performed a phylogenetic analysis to identify such bacteria (Fig. 3E). Different *E. coli* subtypes (e.g., *E. coli* K12, *E. coli* BL21 (DE3), and *E. coli* HB101) showed 100% protein sequence identity with *E. coli* MG1655. Furthermore, *Shigella* exhibited the highest sequence similarity to *E. coli* MG1655, followed by certain species of *Acinetobacter* and *Citrobacter*. Predictions based on this approach are expected to be more accurate for species with high sequence similarity, whereas accuracy decreases for those with lower similarity.

### Factors influencing eARG degradation by *E. coli* MG1655 cell lysate

**Fig 4:**
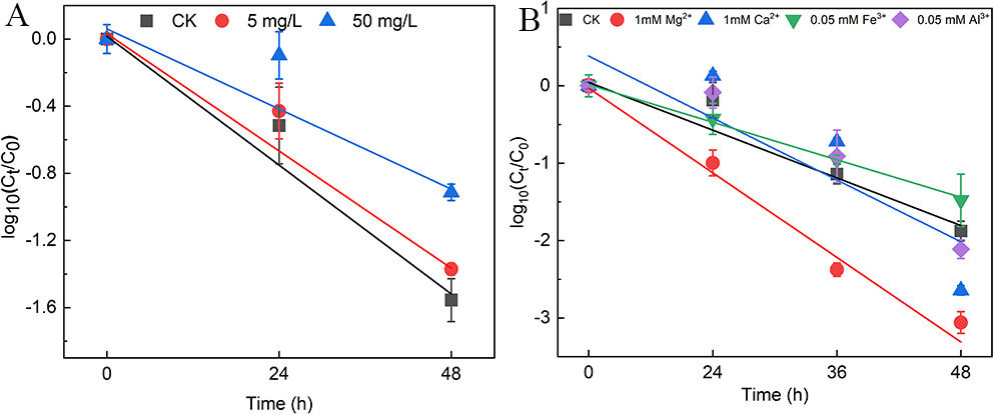
Impacts of environmental factors on the eARG (pUC19) degradation by cell lysate of *E. coli* MG1655. (**A**) Different concentrations of humic acid (0, 5, and 50 mg/L) and (**B**) metal ions (1 mM Mg^2+^, 1 mM Ca^2+^, 0.05 mM Fe^3+^, and 0.05 mM Al^3+^). Data are presented as the mean ± SD (*n* = 3). Lines are the linear regressions of the experimental data. CK group refers to eARG (pUC19) degradation by cell lysate of *E. coli* MG1655 without any addition of humic acid or metal ions.

The *E. coli* MG1655 cell lysate (0.4 mg/mL) was found to degrade eARG by 1.56 log, 1.37 log, and 0.91 log at concentrations of 0, 5, and 50 mg/L of HA, respectively (Fig. 4A). The degradation kinetic constant declined from 0.075 (*R^2^* = 0.96) in the control to 0.066 (*R^2^* = 0.96) and 0.044 (*R^2^* = 0.83) in the treatments with 5 mg/L and 50 mg/L of HA, respectively. Mg^2+^ at environmentally relevant concentrations had a positive impact on eARG degradation by cell lysate (Fig. 4B). The degradation kinetic constant for eARG in the treatment with Mg^2+^ (0.14 h^−1^, *R^2^* = 0.98) was significantly higher than the control without Mg ^2+^(0.085 h^−1^, *R^2^* = 0.84) (Fig. S7), while the degradation kinetic constant decreased in the presence of Fe^3+^ (0.05 mM) and Al^3+^ (0.05 mM), with corresponding values of 0.054 (*R^2^* = 0.95) and 0.071 (*R^2^* = 0.84), respectively (Fig. S7). These results suggest that the nuclease in the cell lysate requires Mg^2+^ ion for activation, while Fe^3+^ and Al^3+^ in the environment may interfere with its activity.

*E. coli* MG1655 has a higher maximum biomass and growth rate compared to its sibling bacteria (Fig. S8; Table S8). Moreover, *E. coli* MG1655 exhibited stronger tolerance to heat, acid, and osmotic stress compared to other sibling strains like *E. coli* JM109, *E. coli* BL21 (DE3), and *E. coli* HB101 (Fig. S8 and S9). Furthermore, the presence of a nuclease inhibitor in the treatment effectively inhibited the degradation of eARGs by the cell lysate of these sibling strains (Fig. S10), and agarose gel electrophoresis results revealed the presence of the *endA* and *recA* genes in the genomes of these bacteria (Fig. S11). These results further revealed that the cell lysates of these sibling strains of *E. coli* may also have a similar mechanism to degrade eARGs.

### Immobilization of *E. coli* MG1655 cell lysate in PAM hydrogel and its usefulness in degrading eARGs in water

**Fig 5:**
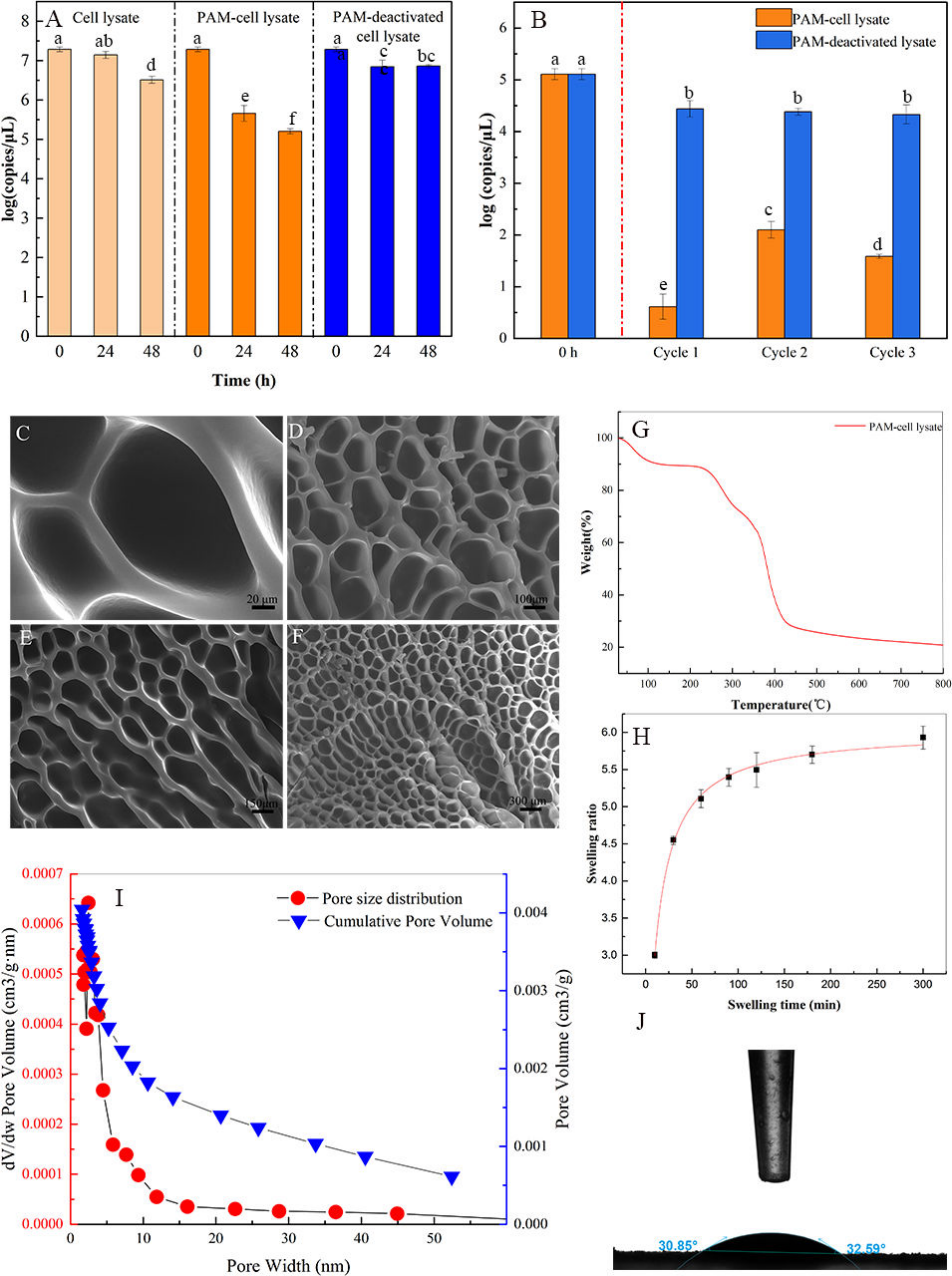
Degradation characteristic of the extracellular *amp^R^* gene on the pUC19 plasmid by immobilized PAM cell lysates and the physicochemical properties of the PAM cell lysates. (**A**) Changes in concentrations (short-amplicon qPCR (178 bp) of *amp^R^* genes under free cell lysate or PAM-cell lysates treatment in ultrapure water. The initial concentration of pUC19 was 0.1 ng/μL. (**B**) Reusability of PAM-cell lysate during continuous three sequential cycles using real wastewater, with each cycle lasting 24 h. Changes in concentrations of the *amp^R^* gene were measured by short-amplicon qPCR (178 bp). The initial pUC19 plasmid copies were set at an environmentally relevant concentration of 10^5^ copies/μL. (**C–F**) SEM images of polyacrylamide hydrogel-immobilized cell lysates. (**G**) Thermogravimetric loss of polyacrylamide hydrogel immobilized cell lysates. (**H**) Swelling kinetic profiles of the immobilized cell lysates. Scatters: experimental data; curves: model fitting with scatters: experimental data, and curves: model fitting with $q_{t}$=$W_{e}^{2}kt/\left(1+W_{e}kt\right)$. (**I**) Pore size distribution and cumulative pore volume of the immobilized cell lysates. (**J**) Contact angles of the immobilized cell lysates with water and toluene. Mean differences were statistically analyzed by one-way analysis of variance (ANOVA), followed by all pairwise comparisons using Student Newman Keuls (SNK) post-hoc procedure to assess the significance of individual variations between the treatment groups. Bars with different letters indicate significantly different (*P* < 0.05).

Notably, the PAM-cell lysate exhibited the highest degradation efficiency, while PAM-deactivated cell lysate showed some ability to reduce the level of *amp^R^* in ultrapure water (Fig. 5A). In the degradation of eARG in real wastewater, the PAM-cell lysate displayed impressive cyclic performance over three consecutive runs, consistently reducing eARG levels (3.0–4.5 logs measured by short amplicons in each cycle) (Fig. 5B). In contrast, the PAM-deactivated cell lysate showed only limited eARG removal by adsorption during the three cycles (< 0.7 log) (Fig. 5B). SEM analysis revealed that the PAM-cell lysate exhibited abundant pore structures, with large pores over 100 μm in size that are regularly distributed (Fig. 5C through F). The material demonstrated good thermal stability, showing only 10.1% mass loss under heating conditions (Fig. 5G), and displayed a hydrophilic surface with a contact angle of 30° (Fig. 5J). Freshly prepared PAM-cell lysate showed uniformly distributed pores ranging in size from 2 to 10 nm in the PAM-cell lysate (Fig. 5I), accompanied by a BET specific surface area of 2.51 m²/g (Fig. 5I). Swelling kinetics revealed a characteristic biphasic profile: an initial rapid burst phase followed by a gradual plateau (Fig. 5H). The fitting parameters of the pseudo-second-order swelling kinetic model for the PAM-cell lysates are summarized in Table S9. Notably, no significant increase in wastewater TOC concentration was observed after three consecutive cycles, indicating minimal leaching of the cell lysate into the aqueous environment (Fig. S12).

## DISCUSSION

### *E. coli* MG1655 cell lysate effectively degraded eARG and greatly reduced its transformation frequencies

The present study demonstrated that exposure to *E. coli* MG1655 cell lysate led to a concentration-dependent eARG degradation, as evidenced by the analysis of short and long amplicons qPCR. As the length of the amplicon decreased, the potential targets for damage within the DNA also decreased, resulting in a higher removal kinetics constant for long amplicon qPCR compared to short amplicon qPCR (43, 44). The degradation rate of the eARG, as determined by qPCR, underestimated the loss of transforming frequencies. This discrepancy may be attributed to damage occurring at reactive sites outside the *amp^R^* genes, which can inhibit successful transformation (27, 45). Therefore, complete removal of eARGs is not necessarily required; a limited log-scale eARG degradation by the cell lysate is sufficient to essentially eliminate their transformability, thereby effectively mitigating the risk of ARG dissemination in real wastewater treatment systems. Moreover, *E. coli* MG1655 cell lysate does not exhibit selectivity for the degradation of any of the four nucleotides (Fig. 2A through D), suggesting a broad impact on DNA integrity. These findings verify that *E. coli* MG1655 cell lysates can effectively degrade eARG and greatly reduce its transformation efficiency.

### DNase I in *E. coli* MG1655 cell lysate predominantly carried out the degradation of eARG

*E. coli* MG1655 cell lysate is thermally unstable and can non-selectively degrade the double-stranded forms of eARGs (Fig. 2E; Fig. S6), suggesting the existence of DNase. It is unlikely that DNase II is present in the cell lysate of *E. coli* MG1655, as DNase II is typically localized in lysosomes of the eukaryotes and requires an acidic pH for optimal activity (32). EDTA, responsible for the inhibition of DNase I, efficiently inhibits eARG degradation, confirming our hypothesis that DNase I was present. It is theoretically feasible to obtain DNase I, encoded by *endA*, by lysing *E. coli* MG1655 and its sibling strains. Our results further confirm that EPS itself does not efficiently degrade eARGs, effectively excluding significant DNase I activity within the EPS fraction (Fig. 3A). The non-specific cleavage activity of DNase I against double-stranded DNA is not limited to the pUC 19 plasmid but extends to other vectors like RSF1010 and pCLT, highlighting its broad-spectrum degradation capability and application potential. Although *endA* is a known nuclease gene in *E. coli*, the key proteins involved in nuclease expression and secretion may vary among strains, highlighting the need for further investigation. Despite inherent imprecision, phylogenetic analysis provides useful guidance for prioritizing strains with the greatest potential for DNase I production, especially among these closely related species.

The inhibition effect of HA on eARG degradation can be attributed to the interactions between HA and DNases (46). Previous studies have shown that extracellular DNA bound to HA is more resistant to DNase I compared to free DNA and retains the ability to be taken up by competent cells (46). Furthermore, in complex environments, enzymes are tightly associated with HA, and their enzymatic activity and bioavailability can be reduced or even eliminated due to structure deformation (47, 48). In contrast, environmentally relevant concentrations of Mg^2+^ and Ca^2+^ had promoting effects on eARG degradation by *E. coli* MG1655 cell lysate (Fig. 4B), which was consistent with the known activation of DNase I by these divalent ions (49). The enhanced degradation observed in our study supports the hypothesis that DNase I, which constitutes the primary nuclease in the *E. coli* MG1655, contributes to eARG degradation. All these pieces of evidence collectively suggest that eARG degradation by *E. coli* MG1655 cell lysate is likely due to the presence of DNase I in the intracellular environment rather than secreting DNase I extracellularly. The plasmids are first degraded and then broken down into DNA fragments and smaller molecules that cannot be detected by gel electrophoresis due to the non-selective degradation characteristics of DNase I on eARG.

### Application potential of PAM-cell lysates to degrade eARGs in water

Surprisingly, eARG degradation efficiency by PAM-cell lysate was higher than the equivalent degradation by dissolved cell lysate (Fig. 5A). PAM-cell lysate possesses abundant channels and mesopores, as confirmed by SEM and pore size distribution analysis, allowing substrate molecules to freely move within the hydrogel. Furthermore, the network structure of PAM-cell lysate, enriched with hydrophilic material, enhances hydrophilicity, as evidenced by swelling kinetic profiles and contact angle results. This markedly reduced diffusion resistance within the hydrogel, allowing eARGs to penetrate the three-dimensional matrix instead of being confined to surface reactions. This deep penetration enabled bulk interaction with the encapsulated cell lysate (35), thereby enhancing overall degradation efficiency. Moreover, during the PAM-cell lysate treatment process, there might exist a dynamic coupling of adsorption and degradation, which contributes to the robust performance of PAM-cell lysates in removing eARGs.

The prevalence of eARG in WWTP is a critical concern, which constitutes the dominant form of eARGs in final effluents, with reported concentrations of 8.6 μg/L in secondary effluent (50, 51). Therefore, inhibiting the transformation and uptake of eARGs presents a crucial strategy for curbing the dissemination of antibiotic resistance in WWTP effluent. Although this cell-free enzymatic approach cannot prevent the live-cell-mediated transfer of iARGs, it remains a critical strategy for reducing antibiotic resistance by specifically inhibiting the transformation and uptake of the highly abundant eARGs in WWTP effluent. Our approach of immobilizing *E. coli* MG1655 cell lysates within PAM hydrogels directly addresses this challenge, which effectively removed 99.9% of *amp^R^* across three consecutive treatment cycles (Fig. 5B). The immobilization matrix provides a stabilizing and protective microenvironment that preserves lysate activity over an extended duration. This cell-free, enzymatic strategy offers a targeted method for DNA degradation, presenting a distinct advantage over conventional broad-spectrum disinfection technologies, such as chlorination, ozonation, and UV-based advanced oxidation processes, which can generate toxic byproducts. Furthermore, PAM-immobilized lysate holds key advantages over other emerging biotechnologies. Unlike synthetic microbiomes or engineered live cells (e.g., using *D. radiodurans* or *S. oneidensis*), our cell-free system eliminates biosafety risks associated with the release of live or engineered bacteria and simplifies operation by avoiding complex microbial community management. The system leverages the natural DNase I activity of a non-modified, industry-relevant host, *E. coli* MG1655. This strain was selected for its rapid growth (maximum specific growth rate of 0.99 h⁻¹) and resilience to osmotic and acidic stresses, properties that enable cost-effective and scalable lysate production. The process thrives in standard cultivation conditions and foregoes the need for expensive purification or exogenous cofactors. Therefore, although the system requires a high concentration of crude lysate (e.g., 0.4 mg/mL), the low production cost of the host strain, coupled with the enhanced stability and high reusability provided by the PAM matrix, substantially reduces its long-term operational costs. Consequently, this technology represents a significant and promising advancement over current treatment technologies.

The hydrophilic hydrogel network, evidenced by a water contact angle of 30° and a swelling ratio exceeding 200%, reduces diffusional resistance. This property facilitates direct integration into effluent streams without requiring infrastructural modifications, highlighting its potential for real-world applications. Impressively, the system remained active at high levels of HA (50 mg/L) without contributing to a rise in effluent TOC, indicating robust performance under challenging conditions. To advance this technology from lab validation to practical implementation, several critical challenges must be addressed through future research. These include evaluation of performance across diverse contaminant profiles, optimization of reactor geometry and design, determination of effective operational parameters and dosages, and assessment of efficacy under fluctuating bacterial communities. For instance, implementation of PAM-cell lysate in a continuous-flow reactor could leverage its high-density lysate retention to achieve efficient eARG degradation at shorter hydraulic retention times (e.g., a few hours). Critical steps toward technology maturation involve a comprehensive assessment of long-term hydrogel stability and fouling mitigation. Strategies to mitigate fouling, such as engineering size-exclusive hydrogel composites, incorporating antifouling coatings, or implementing robust pre-filtration, should be further developed. Future work should therefore focus on advanced polymerization techniques, alternative hydrogel matrices (e.g., chitosan and alginate), and lysate purification protocols to enhance stability and address safety. Furthermore, it is critical to recognize that in real-world environments, eARGs are frequently embedded within complex matrices, such as the extracellular polymeric substances (EPS) of biofilms or adsorbed onto solid particles. Assessing the degradation efficacy of the immobilized lysate against such matrix-associated eARGs is therefore a vital step toward its practical, full-scale application. The long-term storage technology for PAM-cell lysate also needs to be developed.

**Fig 6:**
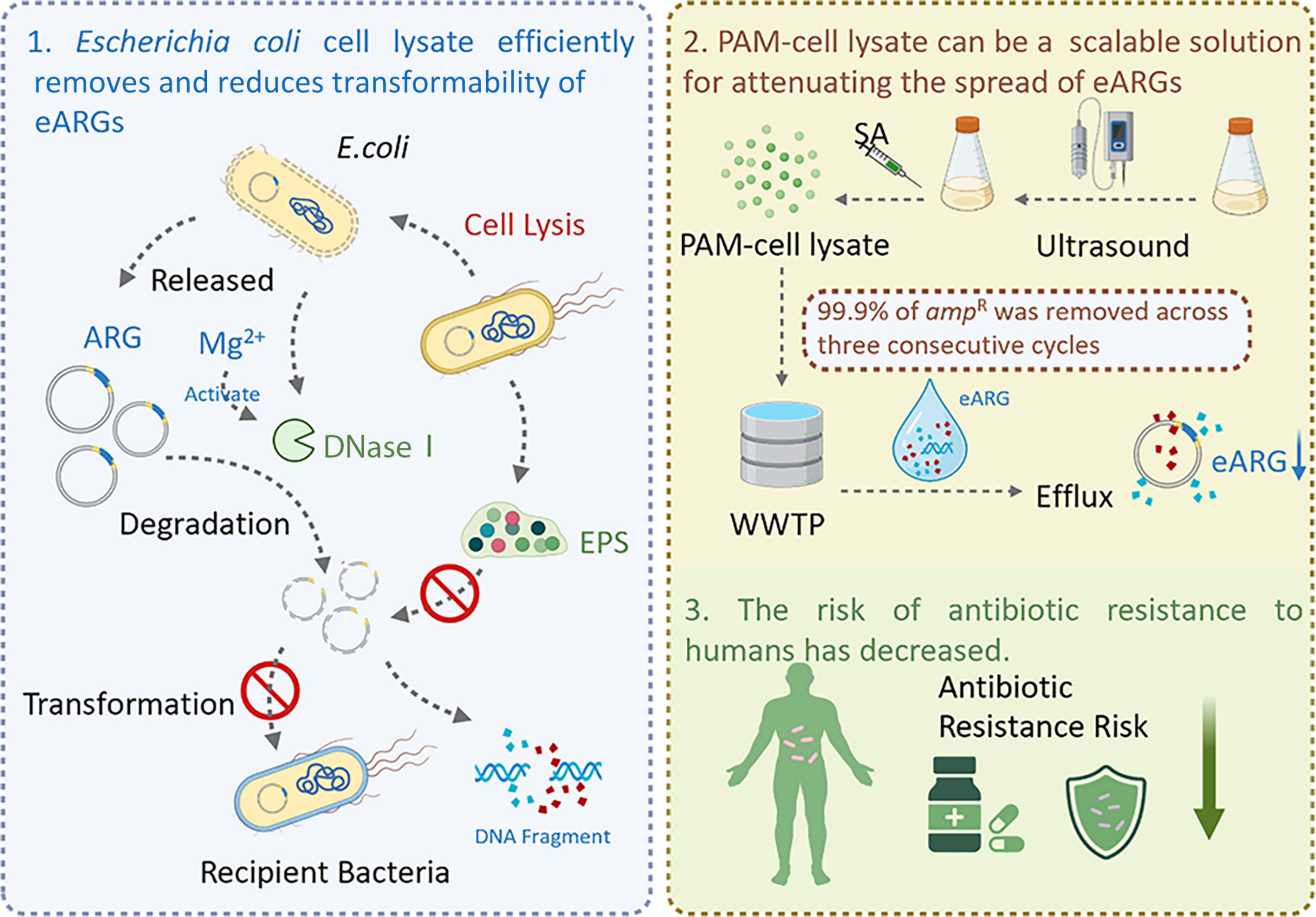
The proposed mechanism through which *E. coli* cell lysate diminishes the transformation of eARGs and reduces the associated risk of antibiotic resistance in humans.

In summary, this work showcases the ability of *E. coli*-derived cell lysate to efficiently eliminate eARGs and inhibit their transformation, a process mechanistically elaborated in Fig. 6. Beyond this, this study underscores the utility of lysate immobilized in a PAM matrix, which exhibits improved reusability and degradation efficiency, as a scalable and eco-friendly technology platform to limit the propagation of antibiotic resistance in water treatment systems. By substantially reducing the environmental prevalence and mobility of eARGs, this strategy effectively mitigates their associated risks to public health.

### Conclusions

This study introduces a novel and scalable strategy to combat eARGs in water by utilizing the natural DNase activity of *E. coli* MG1655 cell lysate immobilized in PAM hydrogels. The lysate effectively degraded plasmid-borne *ampR* and greatly reduced plasmid transformability. In real wastewater, the engineered PAM-cell lysate outperforms free lysate, maintaining over 99.9% of *amp^R^* removal over three cycles. This is possible by the mesoporous structure of the hydrogel, which allows for synergistic adsorption and degradation. Unlike genetically modified or live-cell systems, this approach eliminates ecological risks while offering compatibility with existing infrastructure and cost-effective scalability. Potential improvements to the system may include the incorporation of recyclable components, such as magnetic nanoparticles, to further enhance performance within complex matrices. By combining enzymatic specificity, material innovation, and environmental safety, PAM-cell lysate can be a cost-effective and scalable solution for intercepting the spread of eARGs in water, advancing the development of enzymatic strategies for sustainable water remediation.

## Data Availability

qPCR and other data obtained in this study are available upon request.

## SUPPLEMENTAL MATERIAL

Supplemental material aem.00253-26-s0001.docx Text S1 to S9, Fig. S1 to S12, and Tables S1 to S9.

ASM does not own the copyrights to Supplemental Material that may be linked to, or accessed through, an article. The authors have granted ASM a non-exclusive, world-wide license to publish the Supplemental Material files. Please contact the corresponding author directly for reuse.
