# Supplementary material for: Polyacrylamide hydrogel-immobilized Escherichia coli cell lysate for efficient removal and reduction in transformability of extracellular antibiotic resistance genes in water
Source: Appl Environ Microbiol. 2026 May 6;92(6):e00253-26. doi: 10.1128/aem.00253-26 (PMC13274351; doi:10.1128/aem.00253-26)
Supplement: Supplemental material — Text S1 to S9, Fig. S1 to S12, and Tables S1 to S9. [file aem.00253-26-s0001.docx]

**Polyacrylamide Hydrogel-Immobilized *Escherichia coli* Cell Lysate for Efficient Removal and Reduction in Transformability of Extracellular Antibiotic Resistance Genes in Water**

*Hua Li*^a^*,* *Fangjuan Li*^a^*, Yajie Shi*^a^*, Xinxin Wang*^a^*,* *Xiaomeng Wang*^a^*, Guanyu Zheng*^a,b,*^*,* *Lixiang Zhou*^a,b^*, Barth F. Smets*^c^

^a^*Department of Environmental Engineering, College of Resources and Environmental Sciences, Nanjing Agricultural University, Nanjing 210095, China*

^b^*Jiangsu Collaborative Innovation Center for Solid Organic Waste Resource Utilization, Nanjing 210095, China*

^c^*Department of Biological and Chemical Engineering-Environmental Engineering, Water Center for Water Technology, Aarhus University, Ole Worms Allé 38200 Aarhus C, Denmark*

^*^Corresponding authors.

*E-mail address*: gyzheng@njau.edu.cn (G. Zheng).

**Text S1** Preparation of bacterial culture

*Escherichia coil* MG1655, *Acinetobacter baylyi* ADP, *Pseudomonas pudita* KT2440 and three other *Escherichia coil* ( *E.coli* JM109, *E.coli* BL21(DE3) and *E.coli* HB101) were inoculated in 50 mL of LB Miller broth and incubated overnight at 37℃ with stirring (120 rpm), respectively. Subsequently, the bacterial cell cultures were centrifuged (28℃, 8000 rpm for 10 min) and washed twice with Tris-HCl buffer (pH 7.1,10 mM). After that, the cell pellets were suspended in 20 mL of Tris-HCl buffer and adjusted to a standardized suspension containing 1×10^9^ cells/mL.

The inoculum of *Acidithiobacillus ferrooxidans* LX5 was cultured in modified 9 K medium spiked with 44.2 g/L FeSO_4_·7H_2_O as the energy source. The medium was pre-acidified to 2.5, and then the culture was shaken in a gyratory shaker at 28℃ and 180 rpm for 3 days until a cell density of 10^8^ cells/mL was reached. To obtain a cell density of 10^9^ cells/mL, the supernatant of the culture medium was removed by centrifuging at 8000 rpm for 10 min, and the cell pellet was further resuspended in fresh 9K medium. The final concentration of the *Acidithiobacillus ferrooxidans* LX5 in the 9K medium was 1 × 10^9^ cell /mL.

**Text S2** Comparison of the robustness of *E. coli* MG1655 with its sibling strains

The robustness of *E. coli* MG1655 was evaluated in comparison to its sibling strains (*E. coli* JM109, *E. coli* BL21(DE3), and *E. coli* HB101) under various stressed conditions including acid condition (pH=5.5), high NaCl concentration (30 g/L), elevated Mg^2+^ (10mM) and Ca^2+^ (10 mM) concentrations in LB solution after 12 hours of culture. The initial OD_600_ of each strain in the 100 mL LB culture was kept consistent. Optical density measurements were using a spectrophotometer at 600 nm, and the relative changes in the maximum OD_600_ under the stressed conditions were calculated according to the equation (3).

The relative changes = (max OD _unstressed_- max OD_stressed_)/max OD _stressed_ ×100% （3）

**Text S3 The qPCR analysis of short and long amplicons**

The qPCR reaction was performed on a QuantStudioTM 6 and 7 Flex Real-time PCR system using10 μL of q-PCR reaction solution. Each reaction mixture consisted of 5 μL SYBR Green I Master mix, 2 μL DNase-free water, 0.5 μL forward primer and 0.5 μLreverse primer, and 2 μL purified plasmid template. The temperature cycling protocol for qPCR was as follows: initial denaturation at 95℃ for 5 min, followed by 40 cycles of denaturation at 95^◦^C for 10 s, annealing at 56^◦^C for 15 s, and extension at 72^◦^C for 15 s (for short amplicons) or 30 s (for long amplicons). After the cycling, a melting curve analysis was performed to verify specificity. Each sample was analyzed in duplicate, and the standard curves for eARGs were generated using a 10-fold serial dilution of the plasmids. The standard qPCR curves for eARG were shown in Table S2. The standard qPCR curves for eARG showed excellent model fits (R^2^ > 0.99) and amplification efficiencies of around 100%. All qPCR reactions were performed in duplicate, with a standard deviation of cycle threshold (CT) value of < 0.5 The average copy number was used for subsequent calculations of absolute copy number. The removal efficiency of *amp^R^* can be expressed as log (Ct/C_0_).

**Text S4** Measurements of the concentrations of deoxynucleosides

The concentrations of deoxynucleosides (dC, dG, dA and dT) were simultaneously detected by HPLC (Agilent 1260, USA) coupled with an UV detector. The mobile phase consisted of 90% formic acid (0.1% v/v) and 10% methanol. The flow rate was set to 1.0 mL/min. All four deoxynucleosides were detected at the wavelength of 266 nm. The assignment of each chromatographic peak in the mixture of hydrolyzed DNA samples was determined based on the retention time of each deoxynucleoside. The calibration curves were made using external standards.

**Text S5** Procedure for detecting plasmid pUC19 strand cleavage by gel electrophoresis

Twenty microliters of purified pUC19 plasmid or linearized pUC19 plasmid, prepared by incubating with type II restriction enzyme *EcoRI* at 37℃ for 15 min, were mixed with 1μL of loading buffer, respectively. Then, 10 μL of the mixed samples were loaded onto 1.2% agarose gel with Gold View I. A 1kb ladder mixed with the loading buffer was used to indicate the size of the bands. Gel electrophoresis was conducted in 1×TAE solution at 120V for 40 min. The bands were visualized using a Tanon 1600 imaging system (Tanon Science and Technology Co., China).

**Text S6** The procedures for obtaining EPS from *E. coli* MG1655 bacterial cells and EPS-depleted *E. coli* MG1655 cell lysates

EPS from *E.coli* MG1655 bacterial cells (2×10^9^cells/mL) resuspended in Tris-HCl buffer (pH 7.1, 10 mM) were extracted using the sonicated method (2.7 W/cm^3^, 50 kHz, 15 min) ([Zhou et al., 2020](#_ENREF_12)). After the ultrasonic treatment, the bacterial cells were centrifuged at 11000 g for 20 min. The resulting liquid supernatant was filtered using a 0.22 μm filter to obtain the soluble EPS. The bacterial cell pellet obtained after centrifugation was washed and resuspended in Tris-HCl buffer (pH 7.1, 10 mM) to obtain the EPS-depleted *E. coli* MG1655 bacterial cells. The cell lysate was prepared from EPS-depleted *E. coli* MG1655 as per the method described in the materials and methods.

**Text S7** Procedure for Colony PCR

Colonies measuring approximately 1 mm in diameter were selected as the DNA template for PCR reaction. Amplification of the genes was carried out using Taq DNA polymerase (TAKARA Premix Taq, EX Taq version). The PCR primers utilized for the amplification of *recA* and *endA* genes are shown in Table S5. The thermal cycling parameters included an initial denaturation step at 98 °C for 5 minutes, followed by 30 cycles of denaturation at 94 °C for 30 s, annealing at 58 °C for 30 s, and elongation at 72 °C for 15 s, with a final extension step at 72 °C for 5 minutes. PCR products were visualized on a 1% agarose gel in 1× TAE buffer to verify the specificity of the primers and the existence of the target genes.

**Text S8** Fabrication of hydrogel-entrapped cell lysate

Twenty [milliliter](javascript:;) of the prepared *E. coli* MG1655 cell lysate or the deactivated cell lysate were added to the flask along with 1.065 g of acrylamide, 0.1g of N,N’-methylene-bis-acylamide (MBA), and 0.122 g of K_2_S_2_O_8_.The solution in flask was purged with N_2_ for 5 min. After that, the solution was gently stirred for homogenization under nitrogen, and then placed in a water bath until the reaction mixtures completely gelled. The fresh hydrogel was soaked in distilled water for 48 h to remove the unreacted components. The washed hydrogel was then dried in a freeze drier (Scientz-10N, Ningbo Xinzhi biotechnology co., LTD, Zhejiang, China). The resulting hydrogel cell-lysate composite (denoted as PAM-cell lysate and deactivated PAM-cell lysate) was curshed into the small particles and stored at -20℃ for subsequent use. An initial pre-washing (rinsing) step for the hydrogel is necessary before use to ensure no initial leakage of organic matter into the receiving environment water bodies.

**Text S9** Sudy of swelling characteristics

For the study of swelling kinetics, the dried PAM-cell lysate after weighing (W_0_) (0.005g) was immersed in 1 mL of distilled water at room temperature. The swollen samples were weighed (W_t_) after removing excess surface water with filter paper at various time (10 min, 30 min, 1h, 1.5h, 2h and 3h). The experiments were conducted in triplicate. Swelling experiments were continued until the PAM-cell lysate reached its equilibrium swelling value (W_e_). The swelling ratio (S_t_) and equilibruim swelling ratio (S_e_) was determined by using the following equations.

The swelling kinetics of the samples were obtained by directly fitting the swelling data using the following pseudo-second order model (3), where ks is the rate constant of swelling in unit of g (gel)/g (water)🞌 min.

S_t_=$\frac{Wt-W0}{W0}$ (1)

Se=$\frac{We-W0}{W0}$ (2)

$S_{t}$=${W_{e}^{2}kt}/{(1+W_{e}kt)}$ (3)

**
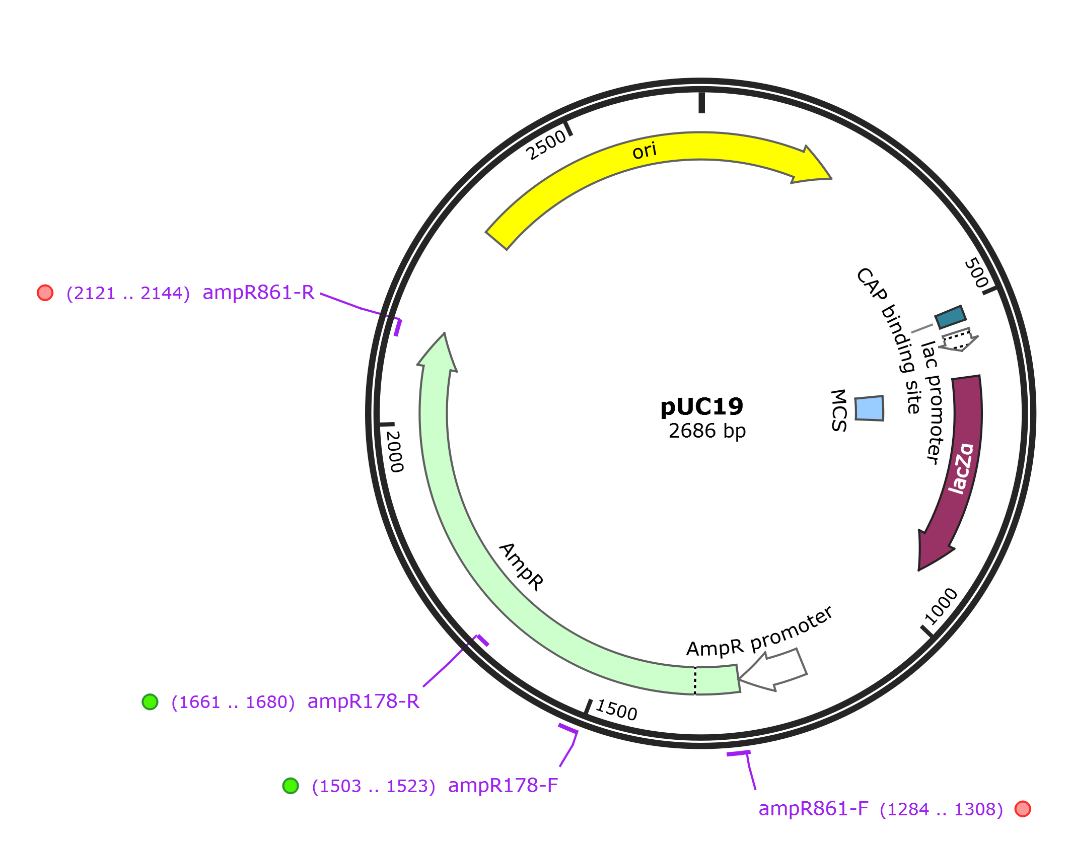
Fig. S1**

**Fig. S1** The structure of plasmid pUC 19 plasmid (2686bp) and the position of the target qPCR amplicons (i.e., 178 and 861 bps of *amp^R^* gene). The complete genome sequence of pUC19 is available at https://www.addgene.org/browse/sequence/74677/

**Fig. S2**


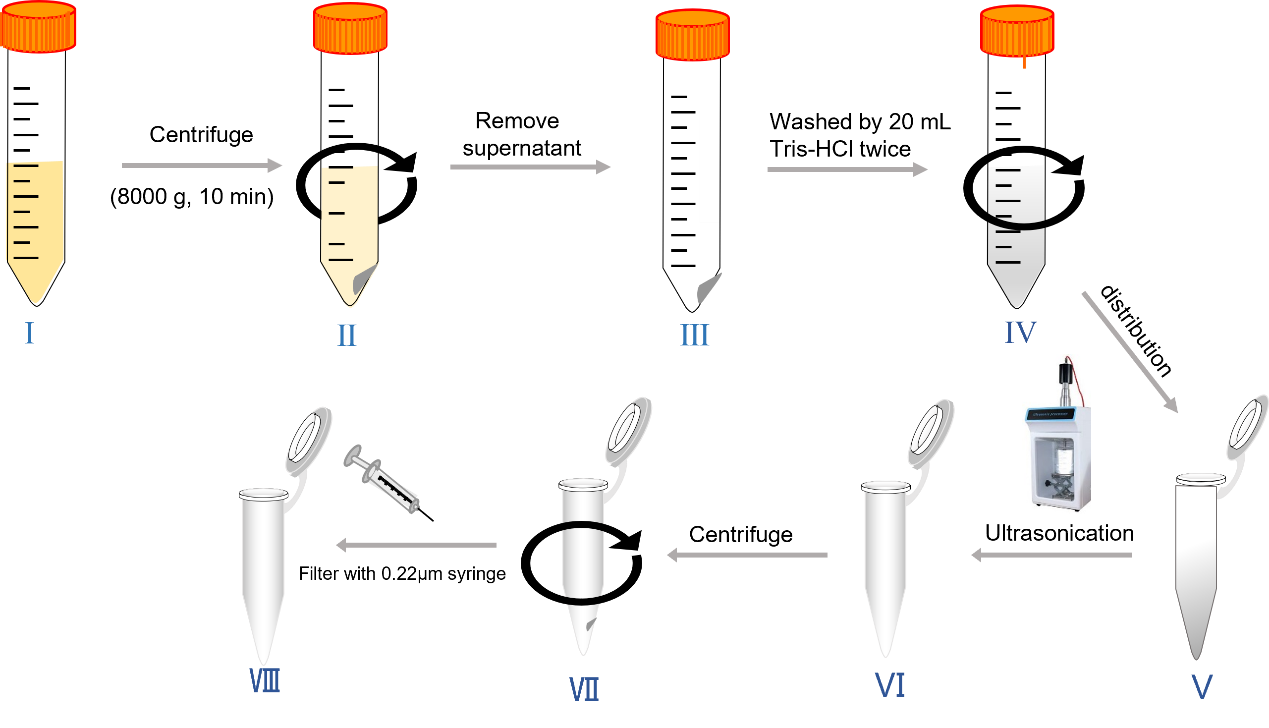


**Fig.S2** The process of cell lysate preparation

**Fig. S3**


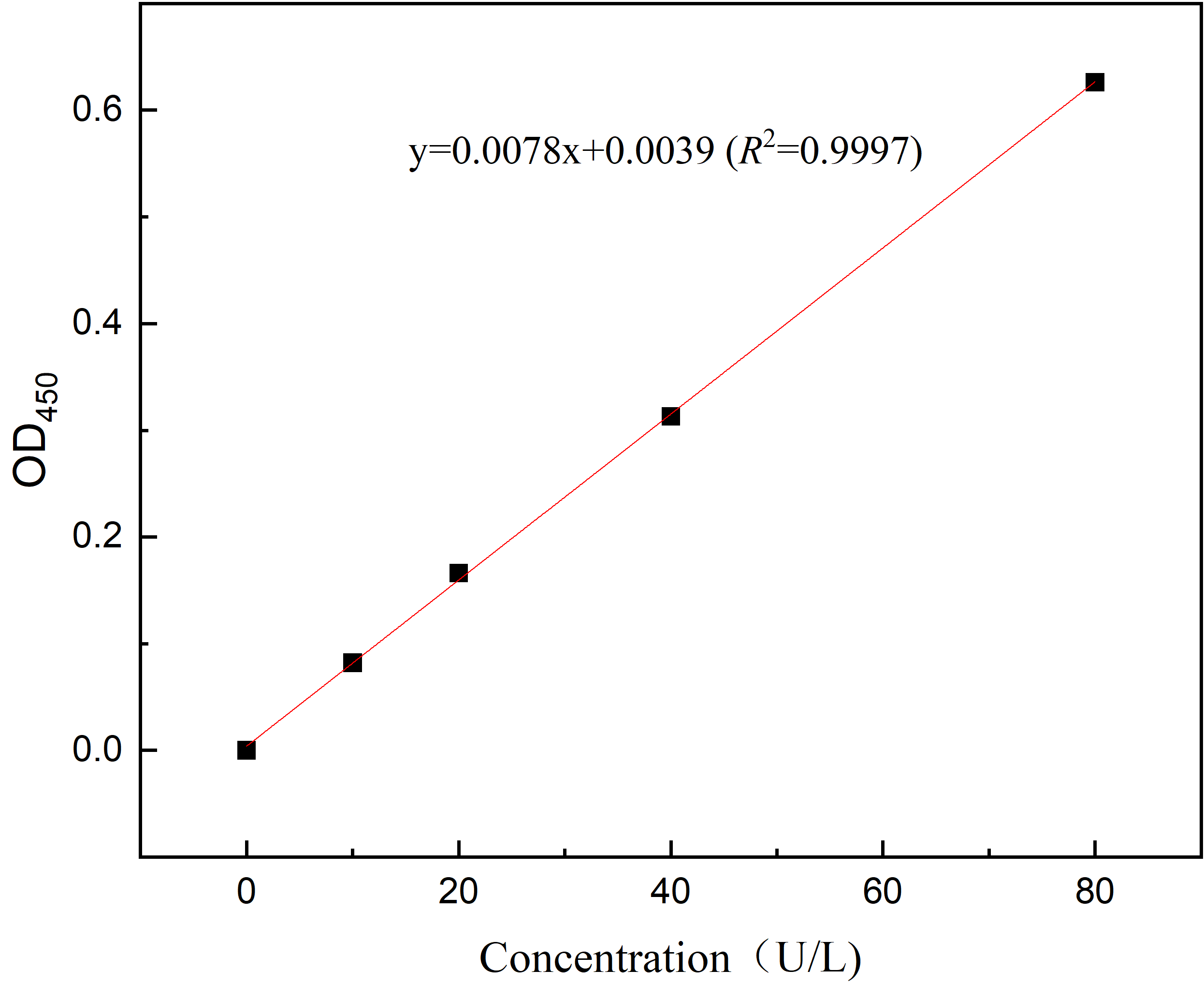


**Fig. S3** Standard curve of Dnase I concentration.

**Fig. S4**


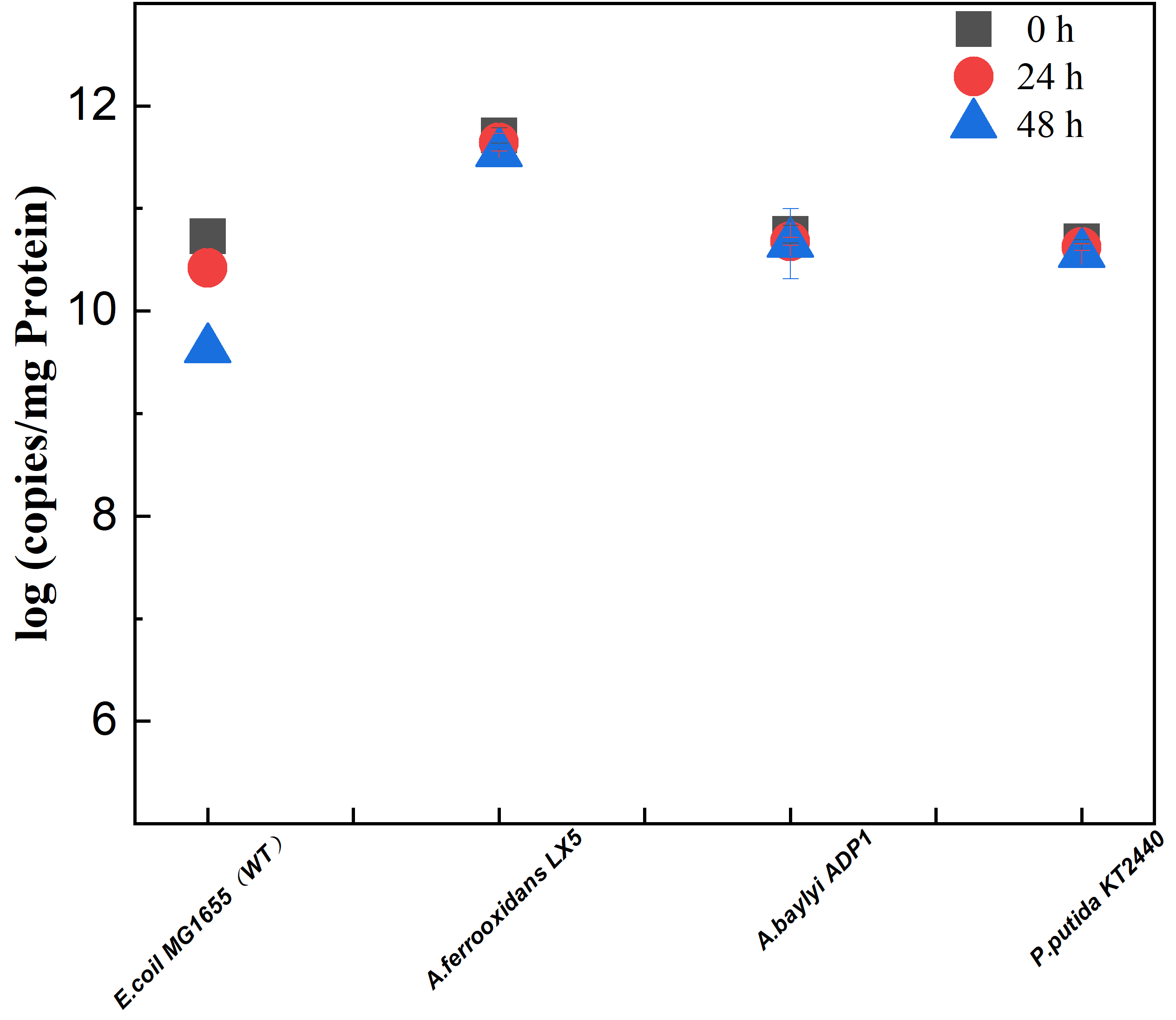


**Fig. S4**. Changes in logarithmic concentrations of *amp^R^* gene (copies) per unit protein concentration of lysates (mg) of four bacterial cells (*E. coli* MG1655, *A. baylyi* ADP1, *P. pudita* KT2440 and *A. ferrooxidans* LX5). The copy number of the *amp^R^* gene was measured by short-amplicon qPCR and the protein concentration was determined by BCA method. Initial concentrations of pUC19 plasmid was 0.1 ng/μL. Protein concentrations of cell lysates of different bacteria were shown in Table S2. Data represent mean values ± S.D (n =3).

**Fig. S5**


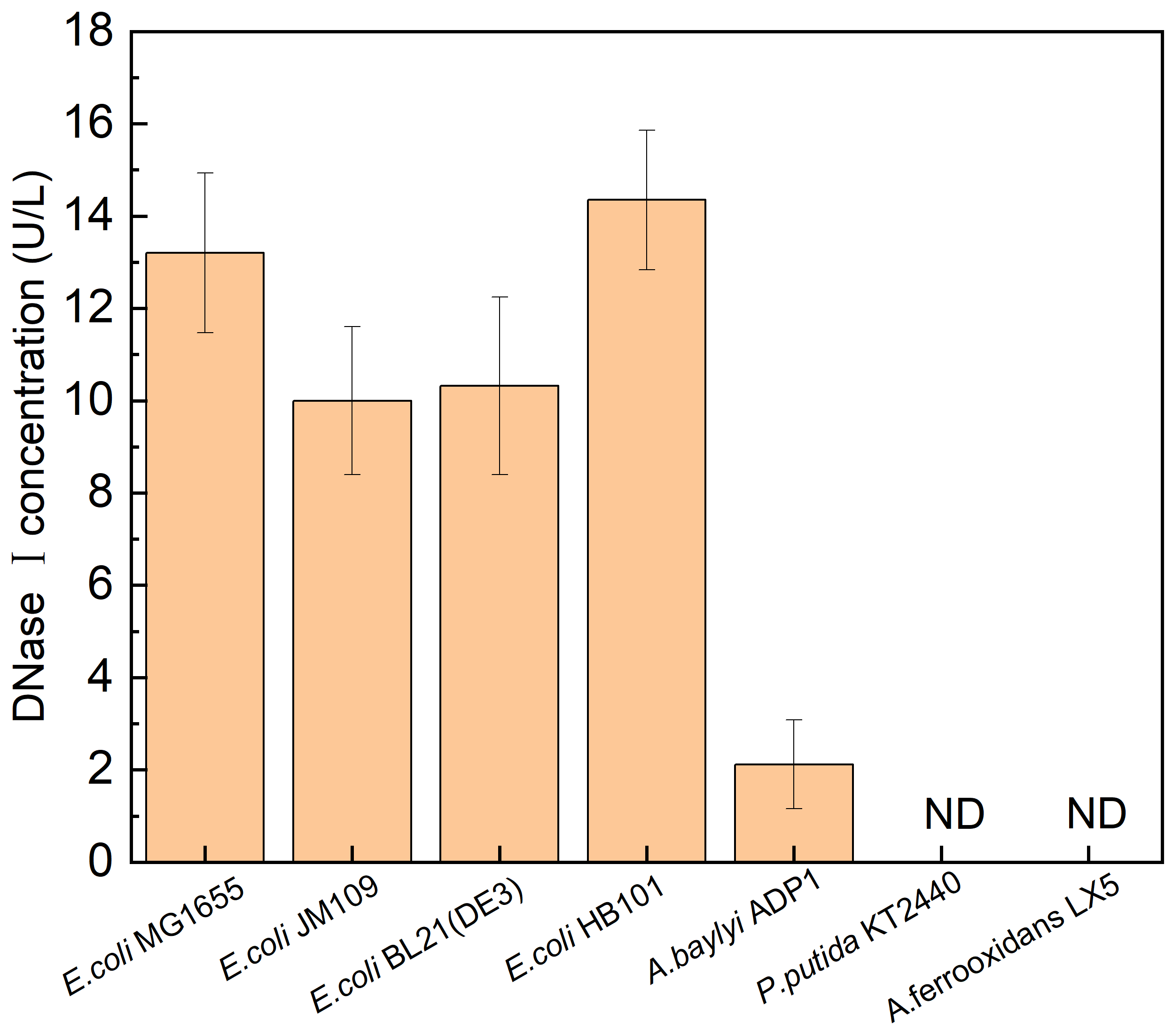


**Fig. S5** DNase I concentration determined in the cell lysate of various bacteria (*E. coli* MG1655, *E. coli* JM109, *E. coli* BL21(DE3), *E. coli* HB101, *E. coli* MG1655, *A. baylyi* ADP1, *P. putida* KT2440 and *A. ferrooxidans* LX5). Cell lysate was prepared by ultrasonic splitting of 10^9^ cells/mL of the bacteria based on the previous method.

**Fig. S6**


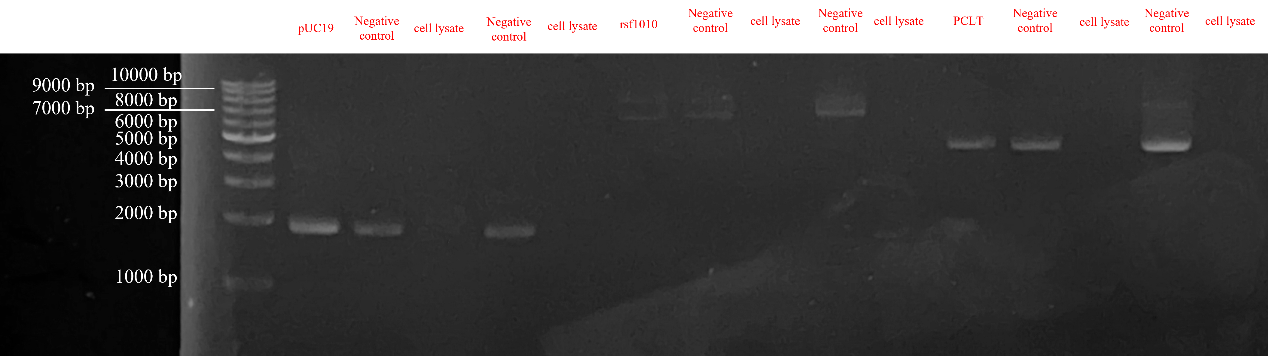


**Fig. S6** Effect of cell lysate of *E. coli* MG1655 on the degradation of ARG-carrying plasmids (i.e. pUC19, rsf1010 and PCLT). The incubation of plasmid alone without adding the cell lysate was set as the negative control. Each sample was subjected to two repetitions on gel.

**Fig. S7**


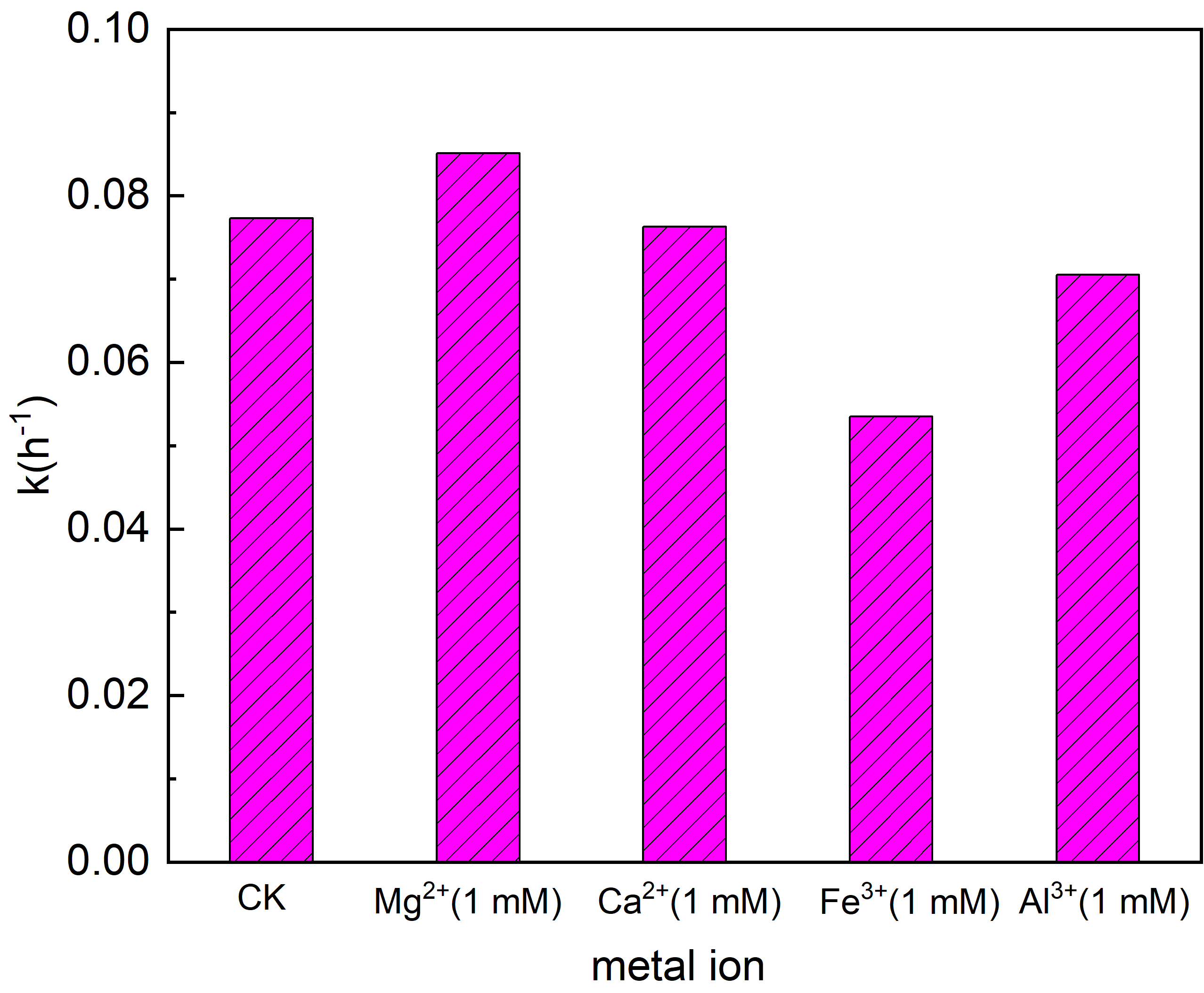


**Fig. S7.** Impacts of co-existence metal ions on degradation rate of *ampR* on pUC19 plasmid by *E. coli* cell lysates. Rate constants were obtained based on the linear regression of the data measured by short-amplicon qPCR. CK group refers to eARG (pUC19) degradation by cell lysate of *E. coli* MG1655 without any addition of humic acid or metal ions.

**Fig. S8**


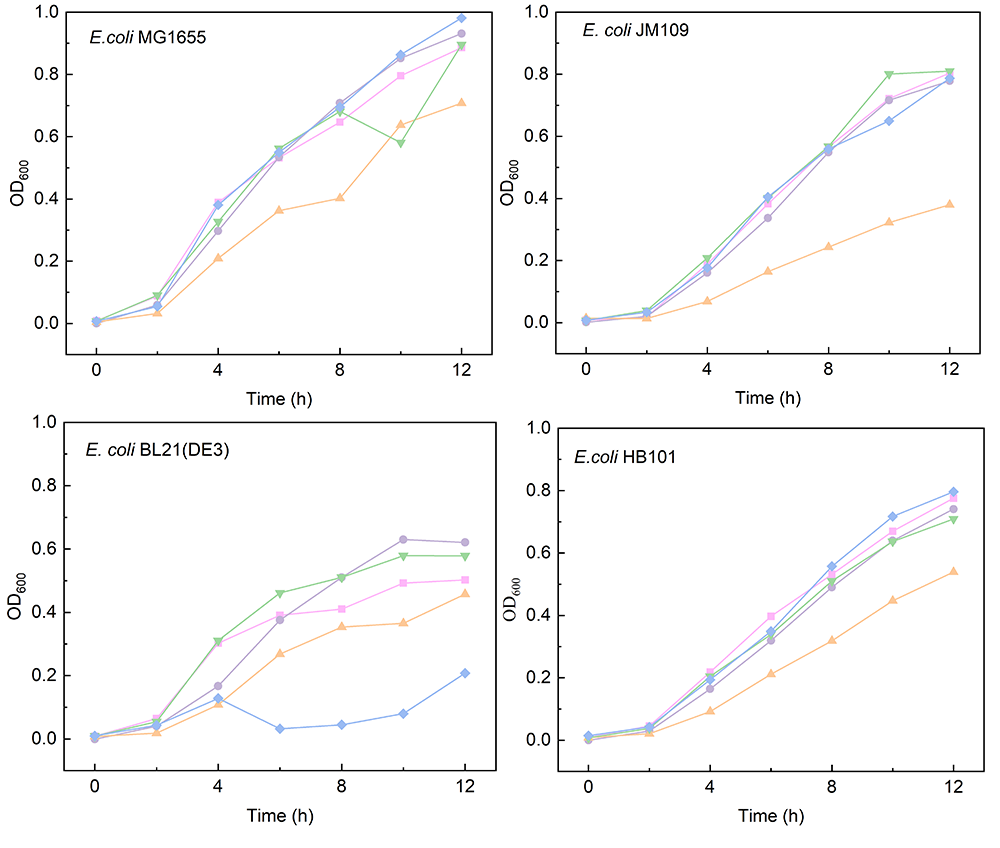


**Fig.S8.** The comparison of *E. coli* MG1655 and its sibling bacteria in growth (
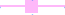
blank control,
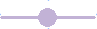
pH = 5.5,
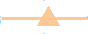
 NaCl = 30 g/L,
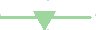
Mg^2+^ = 10 mM,
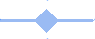
Ca^2+^ = 10 mM).

**Fig. S9**


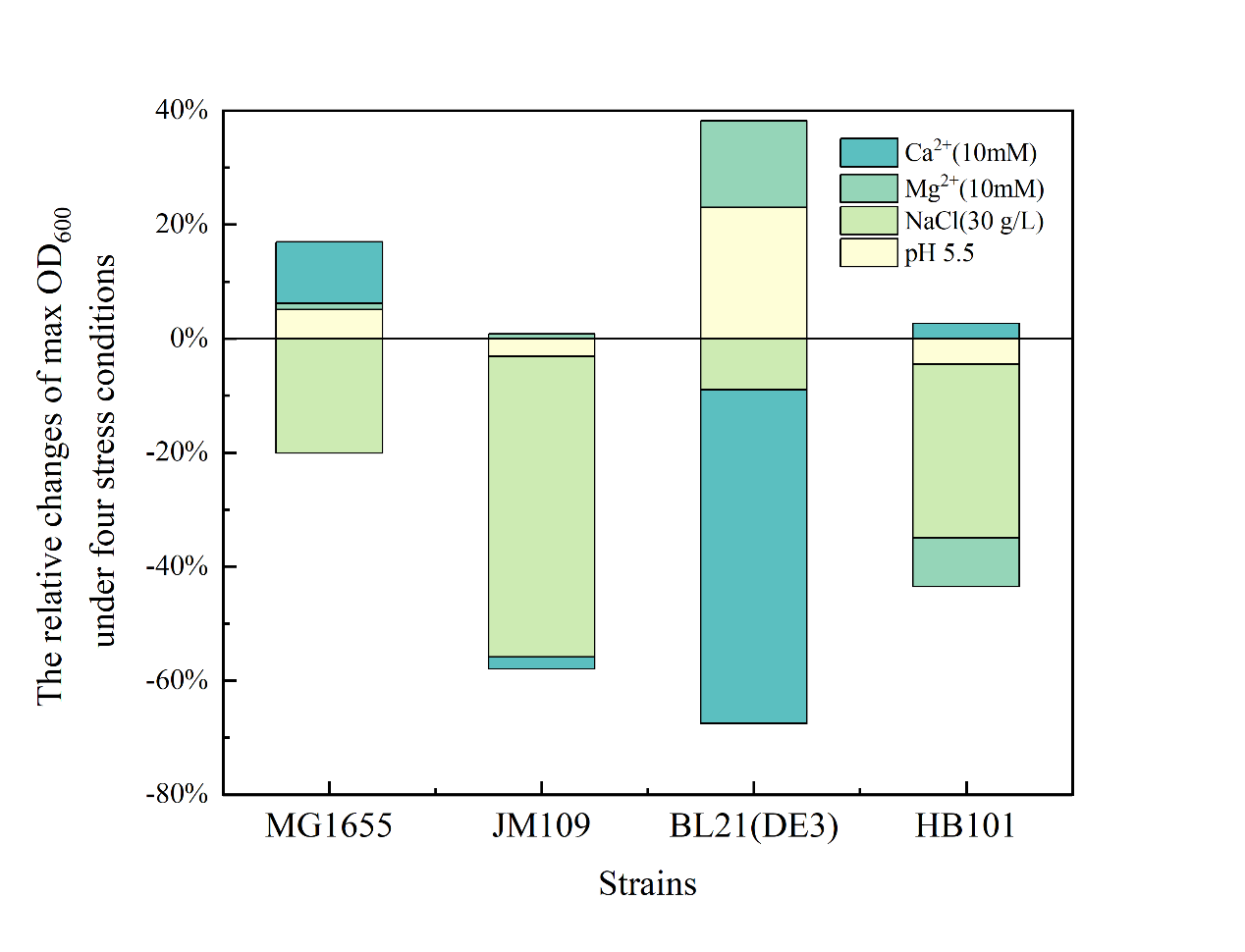

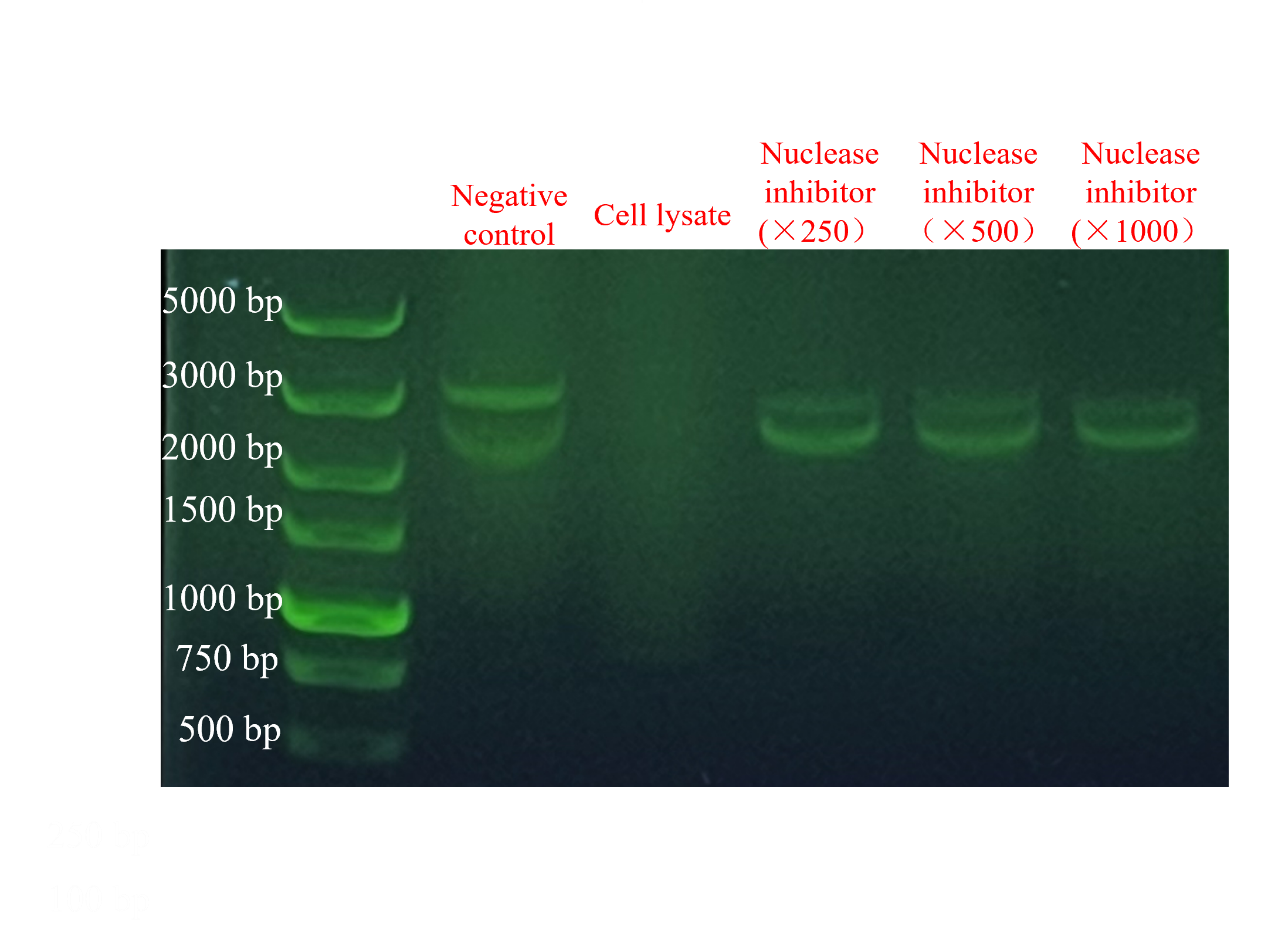


**Fig. S9**. The comparison of *E. coli* MG1655 and three other *E. coli* strains (*E. coli* JM109, *E. coli* BL21(DE3) and *E. coli* HB101) in growth rate and robustness. The fluctuation of max OD of eight strains under three stressed conditions compared to the unstressed condition. Three stressed conditions changed the culture temperature, pH, and the salinity of LB medium, respectively. The relative changes = (max OD unstressed –max OD stressed)/max OD unstressed *100%. Error bars indicate standard deviation (n = 3).


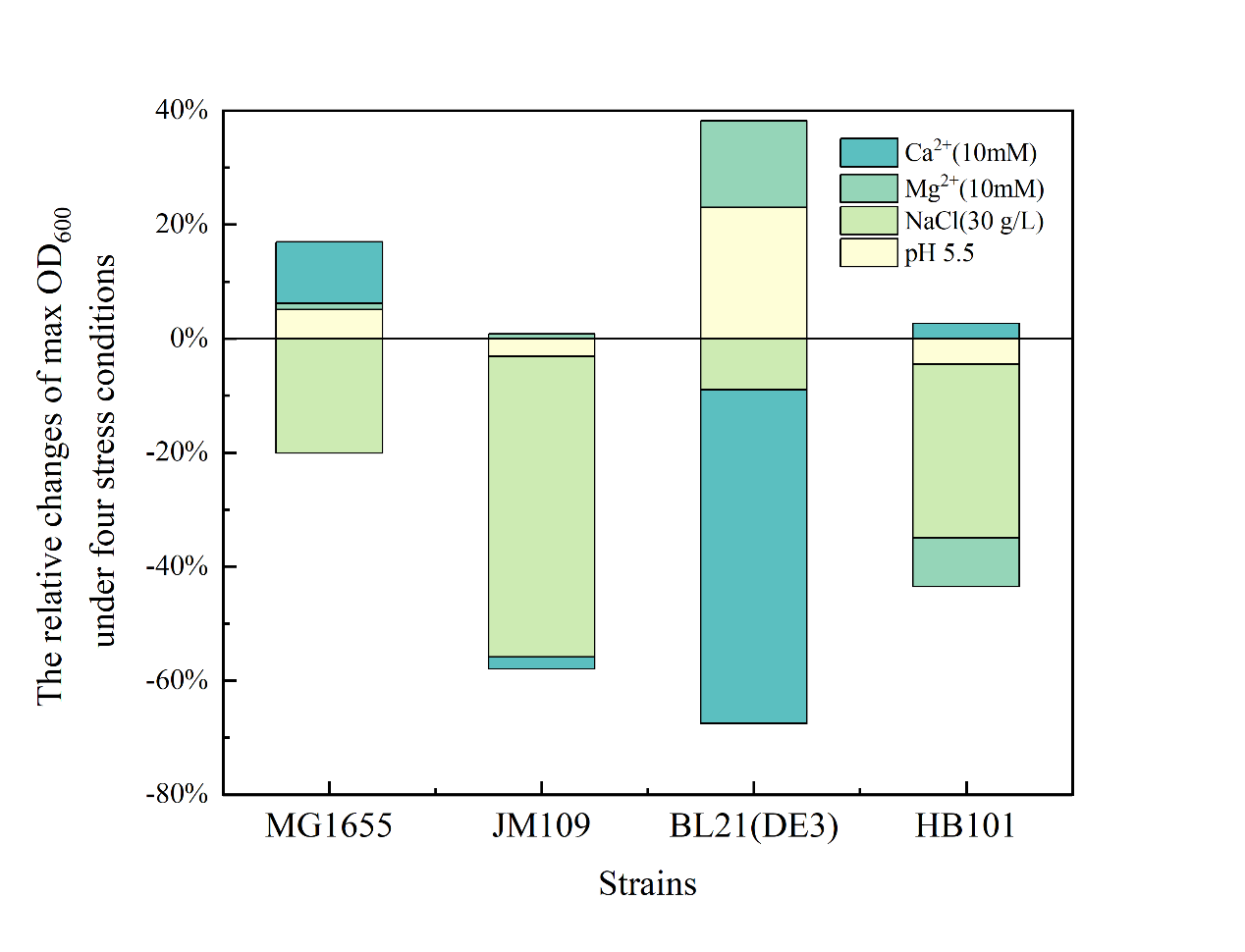


**Fig. S10**


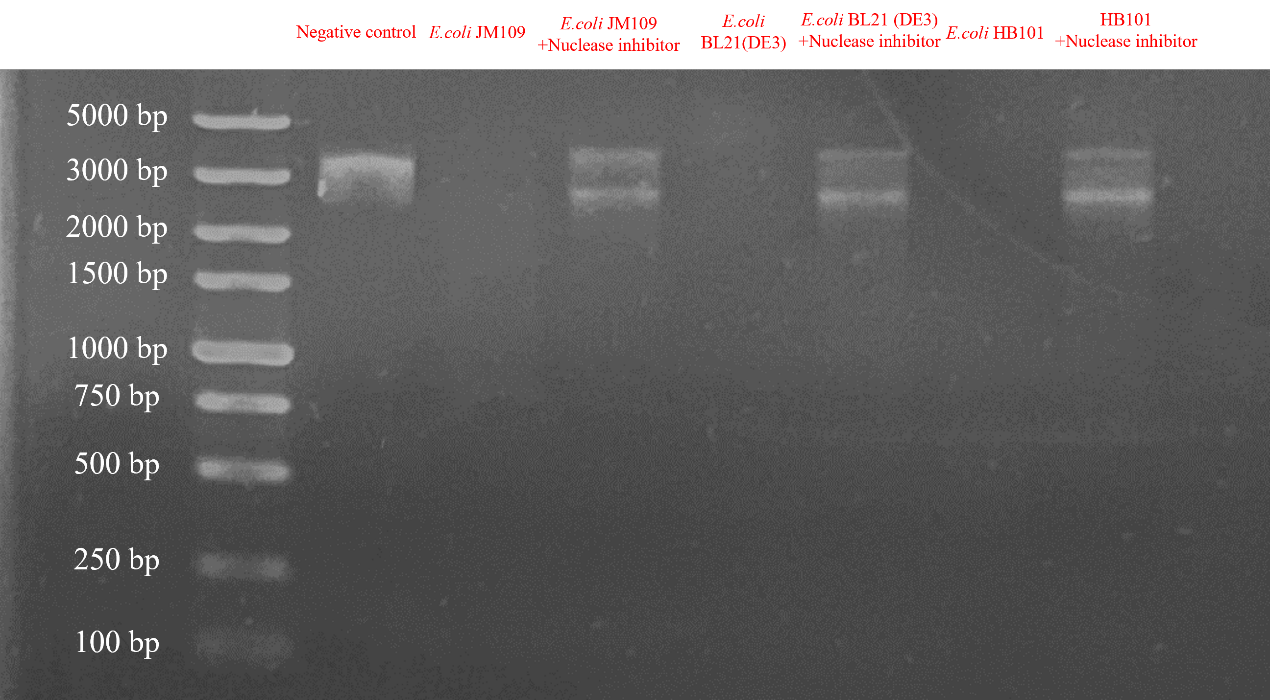


**Fig. S10** Effects of nuclease inhibitor on the degradation of ARG-carrying plasmids co-incubation with cell lysate of *E. coli* JM109, *E. coli* BL21 (DE3) and *E. coli* HB101 for 48 h at 37℃, respectively. The incubation of cell lysate of JM109, BL21 (DE3) and HB101 alone for 48 h at 37℃ was set as the negative control.


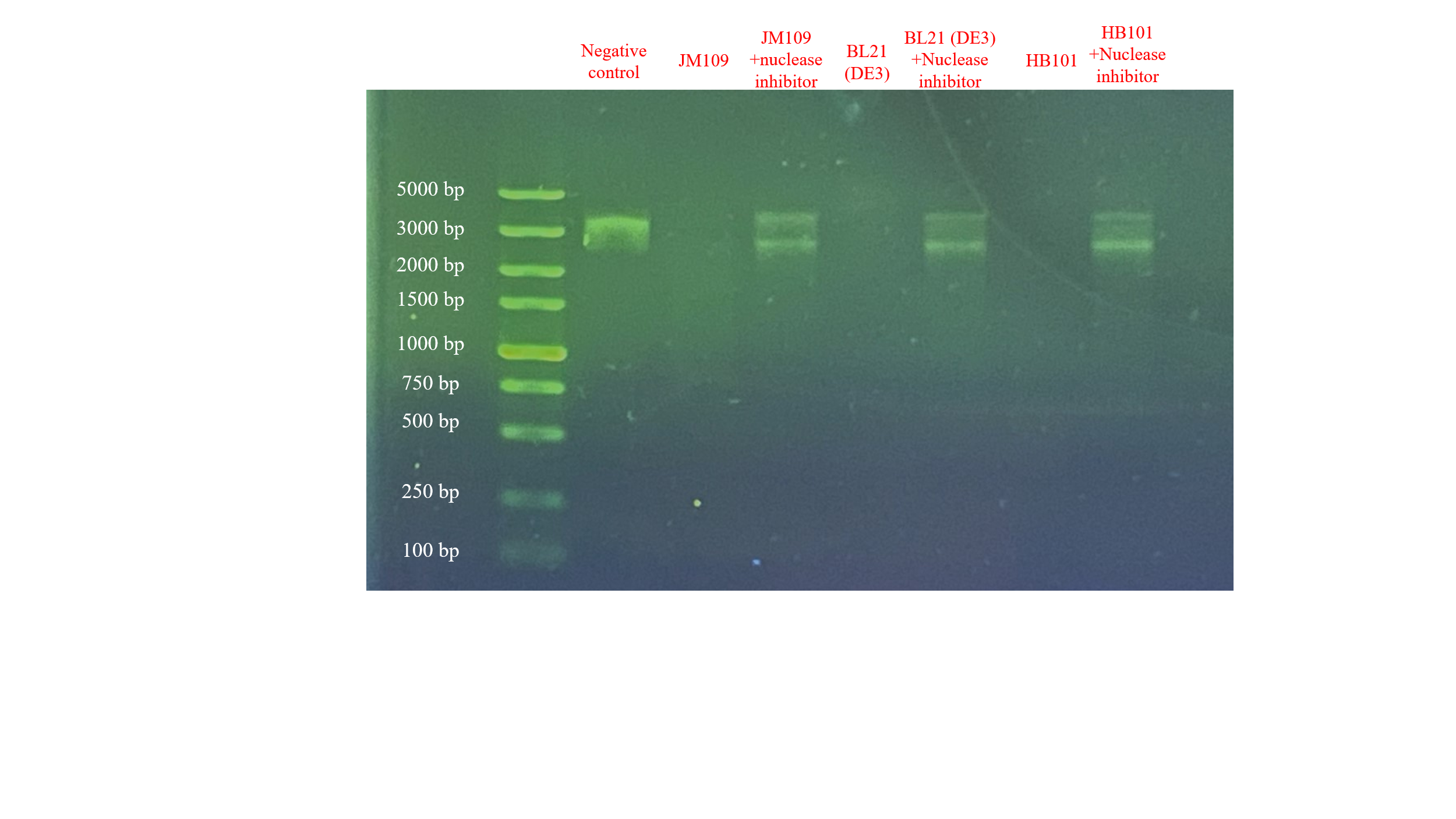


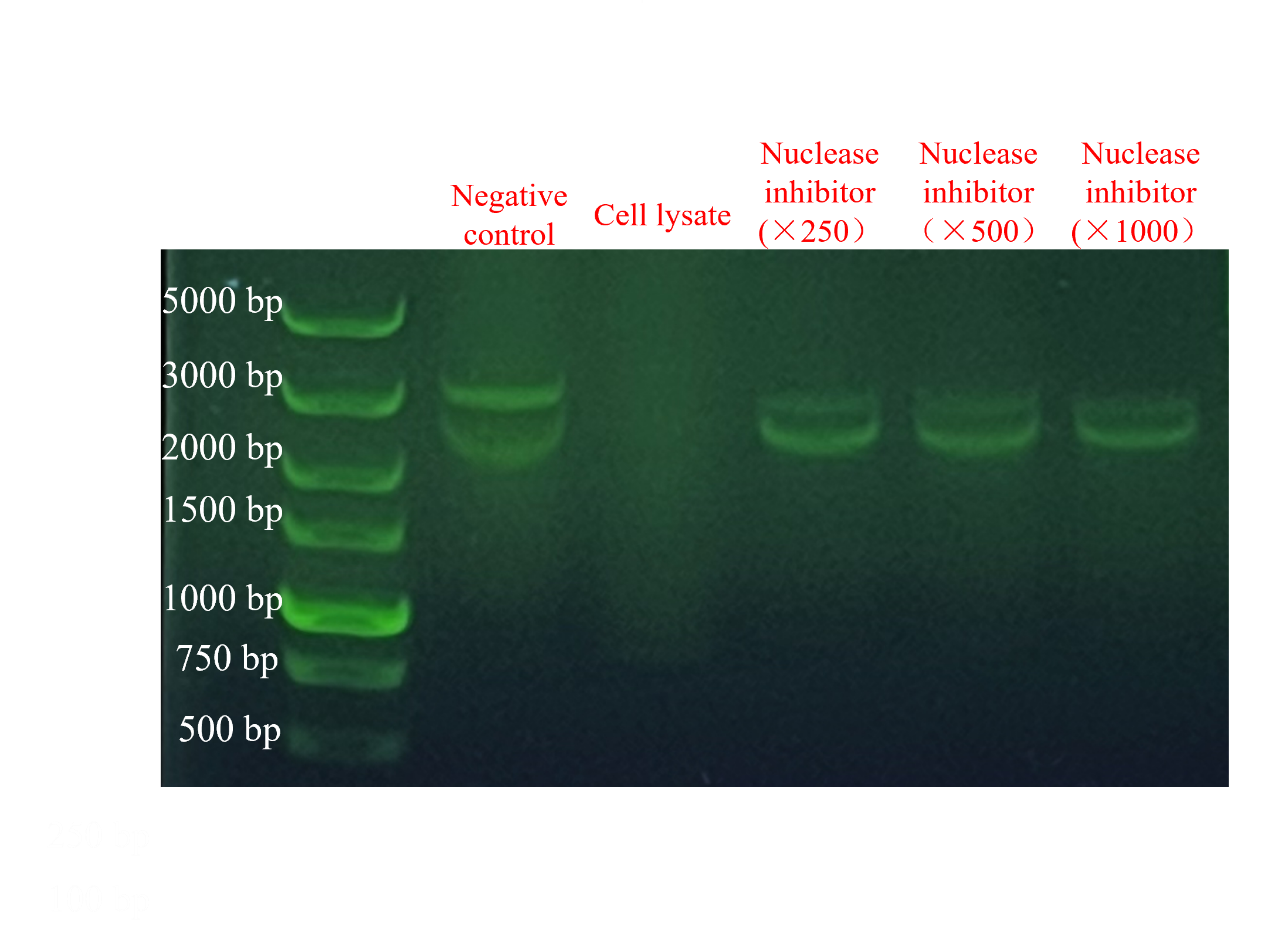


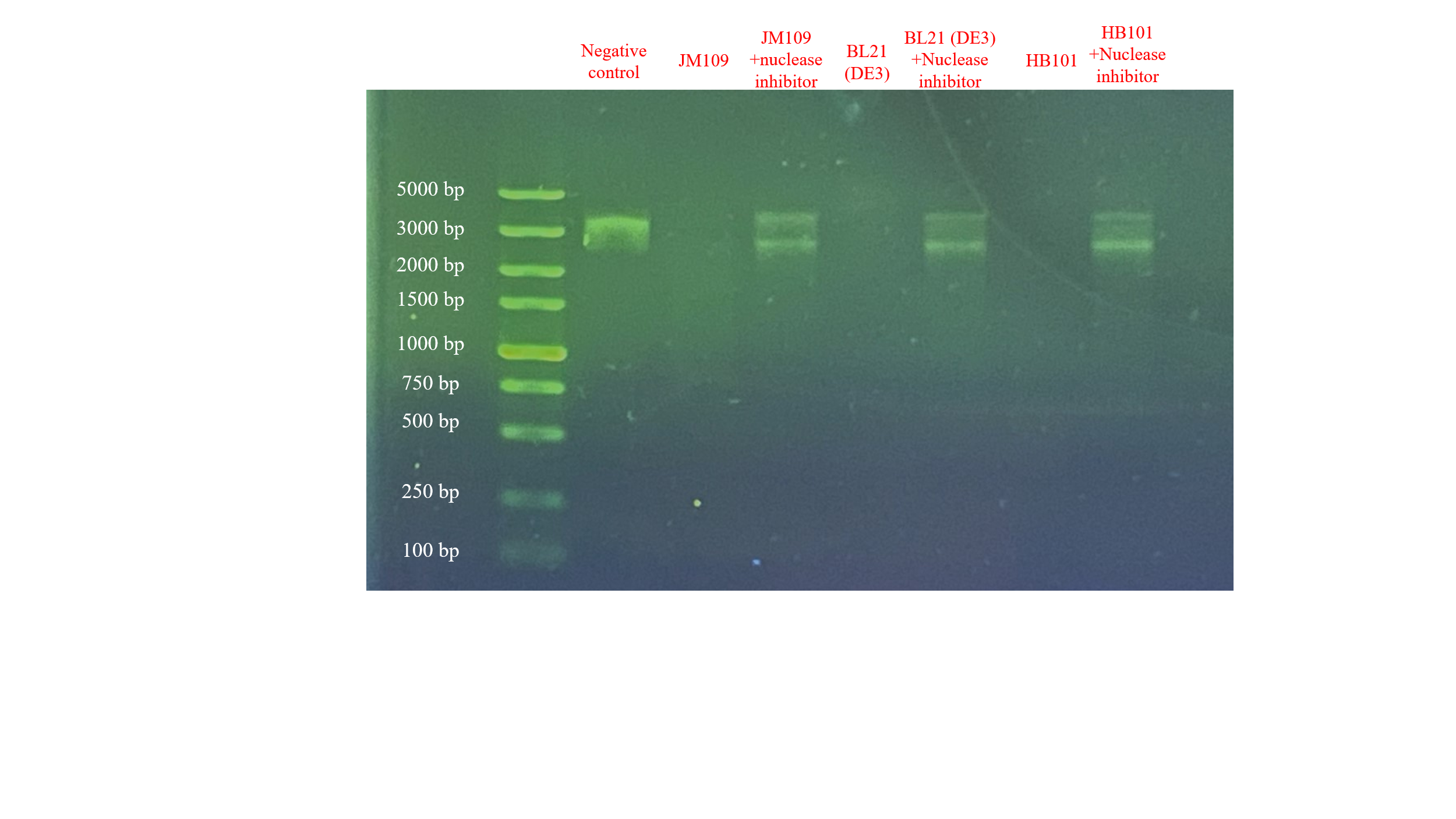


**Fig. S11**


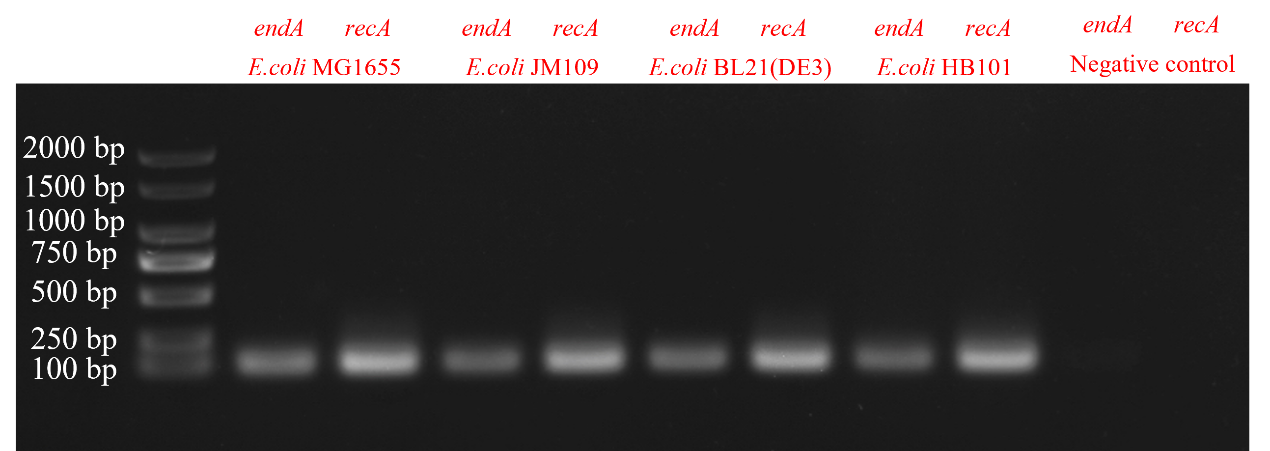


**Fig. S11** Identification of *endA* and *recA* gene in *E. coli* JM109, *E. coli* HB101, *E. coli* BL21 and *E. coli* MG1655 by agarose gel electrophoresis.

**Fig. S12**

**

**

**Fig. S12.** TOC concentrations during three cycles of treatment by PAM-cell lysate.

**Table S1** Protein concentration of bacterial cell lysates determined by BCA method

| Type of the bacteria | Density of the bacteria（cells/mL） | Concentration of the protein（mg/mL） |
| --- | --- | --- |
| *E.coil* MG1655（WT） | 1.99×10^9^ | 0.42 ± 0.0045 |
| *A.baylyi* ADP1 | 1.90×10^9^ | 0.46 ± 0.019 |
| *P.pudita* KT2440 | 1.73×10^9^ | 0.42 ± 0.0040 |
| *A.ferrooxidans* LX5 | 2.05×10^9^ | 0.45 ± 0.0070 |
| *E. coli* JM109 | 1.90×10^9^ | 0.42±0.0070 |
| *E. coli* BL21(DE3) | 1.84×10^9^ | 0.42 ± 0.019 |
| *E. coli* HB101 | 1.92×10^9^ | 0.47 ± 0.019 |

**Table S2** Sequences information of short and long amplicons for the *ampR* gene and the corresponding primers designed by NCBI Primer-BLAST.

| **Amplicon** | **DNA sequence information** | **Primer information** |
| --- | --- | --- |
| amp^R^  short  amplicon（178bp） | CTATGTGGCGCGGTATTATCCCGTATTGACGCCGGGCAAGAGCAACTCGGTCGCCGCATACACTATTCTCAGAATGACTTGGTTGAGTACTCACCAGTCACAGAAAAGCATCTTACGGATGGCATGACAGTAAGAGAATTATGCAGTGCTGCCATAACCATGAGTGATAACACTGCGG | Forward primer CTATGTGGCGCGGTATTATCC  Reverse primer CCGCAGTGTTATCACTCATG |
|  |  |  |
| Long  amplicon（861bp） | ATGAGTATTCAACATTTCCGTGTCGCCCTTATTCCCTTTTTTGCGGCATTTTGCCTTCCTGTTTTTGCTCACCCAGAAACGCTGGTGAAAGTAAAAGATGCTGAAGATCAGTTGGGTGCACGAGTGGGTTACATCGAACTGGATCTCAACAGCGGTAAGATCCTTGAGAGTTTTCGCCCCGAAGAACGTTTTCCAATGATGAGCACTTTTAAAGTTCTGCTATGTGGCGCGGTATTATCCCGTATTGACGCCGGGCAAGAGCAACTCGGTCGCCGCATACACTATTCTCAGAATGACTTGGTTGAGTACTCACCAGTCACAGAAAAGCATCTTACGGATGGCATGACAGTAAGAGAATTATGCAGTGCTGCCATAACCATGAGTGATAACACTGCGGCCAACTTACTTCTGACAACGATCGGAGGACCGAAGGAGCTAACCGCTTTTTTGCACAACATGGGGGATCATGTAACTCGCCTTGATCGTTGGGAACCGGAGCTGAATGAAGCCATACCAAACGACGAGCGTGACACCACGATGCCTGTAGCAATGGCAACAACGTTGCGCAAACTATTAACTGGCGAACTACTTACTCTAGCTTCCCGGCAACAATTAATAGACTGGATGGAGGCGGATAAAGTTGCAGGACCACTTCTGCGCTCGGCCCTTCCGGCTGGCTGGTTTATTGCTGATAAATCTGGAGCCGGTGAGCGTGGGTCTCGCGGTATCATTGCAGCACTGGGGCCAGATGGTAAGCCCTCCCGTATCGTAGTTATCTACACGACGGGGAGTCAGGCAACTATGGATGAACGAAATAGACAGATCGCTGAGATAGGTGCCTCACTGATTAAGCATTGGTAAATGAGTATTCAACATTTCCGTGTCG TTACCAATGCTTAATCAGTGAGGC | \| Forwardprimer ATGAGTATTCAACATTTCCGTGTCG Reverse primer TTACCAATGCTTAATCAGTGAGGC \| \| --- \| |

**Table S3** Standard curve of short and long amplicon of the *amp^R^* gene.

| Gene name | standard curve | *R^2^* | amplification efficiency（%） |
| --- | --- | --- | --- |
| *amp*^R^（178bp） | Ct=-3.3207log_10_(copies/μL)+31.92 | 0.9983 | 100.05 |
| *amp*^R^（861bp） | Ct=-3.3388log_10_(copies/μL)+34.64 | 0.9944 | 99.30 |

**Table S****4** Standard curve of four deoxynucleotides detected by HPLC. Y stands for the peak area and x stands for deoxynucleotide concentration (mg/mL).

| Types of deoxynucleotides | Regression equations | *R*^2^ |
| --- | --- | --- |
| dC | Y=5×10^7^x+16984 | 0.9993 |
| dG | Y=5×10^7^x+10891 | 0.9989 |
| dA | Y=5×10^7^x+11782 | 0.9988 |
| dT | Y=6×10^7^x+10746 | 0.9989 |

**Table S5** PCR primers designed by NCBI Primer-BLAST for *endA* and *recA* genes

| Target gene | Forward primer | Downstream primer |
| --- | --- | --- |
| *endA* | TTGTCTATTGCTGCGGTGGT | GCGTCAGCGTGGACTTTTAC |
| *recA* | GCAGGCACTGGAAATCTGTG | GCCGATTTCGCCTTCGATTTC |

**Table S6** Removal efficiency of four deoxynucelosides (dC, dG ,dA, and dT）in pUC19 plasmid treated by different concentration of cell lysate after 48 hours. Data represent mean values ± S.D (n=3).

| Treatment | Removal efficiency of different deoxynuceloside（%） | | | |
| --- | --- | --- | --- | --- |
|  | dC | dG | dA | dT |
| cell-lysate free water | 7.17±6.83 | 4.91±3.31 | 5.32±2.15 | 3.58±2.05 |
| 0.08 mg/mL | 14.43±2.83 | 9.30±6.95 | 4.58±3.79 | 9.95±6.03 |
| 0.24 mg/mL | 74.60±8.40 | 76.95±7.40 | 85.93±15.40 | 76.05±9.80 |
| 0.40 mg/mL | 89.48±0.43 | 89.74±0.74 | 91.43±0.50 | 91.07±0.50 |

**Table S7** Deoxynuceloside degradation rate constants by different concentration of *E. coli* cell lysate. Concentration of plasmid used here is10 ng/μL.

| deoxynuceloside | Cell lysate concentration (mg/L) | Kinetic rate constant k |
| --- | --- | --- |
| dC | 0.08 | （-1.92±2.28） ×10^-3^ |
|  | 0.24 | （-27.38±5.10）×10^-3^ |
|  | 0.40 | （-55.83±9.08）×10^-3^ |
| dG | 0.08 | （-1.32±2.70） ×10^-3^ |
|  | 0.24 | （-44.46±16.91）×10^-3^ |
|  | 0.40 | （-58.64±13.73）×10^-3^ |
| dA | 0.08 | （-0.33 ± 1.90） ×10^-3^ |
|  | 0.24 | （-43.86±18.07）×10^-3^ |
|  | 0.40 | （-65.15±18.16）×10^-3^ |
| dT | 0.08 | （-1.52±2.32） ×10^-3^ |
|  | 0.24 | （-44.26 ± 18.04）×10^-3^ |
|  | 0.40 | （-63.55±17.09）×10^-3^ |

**Table S8** The detailed number of specific growth rate (h^−1^) and max OD of the *E.coli* MG1655 and its sibling strains cultured in the un- stressed condition (LB, pH 7, 37 ◦C).

| **Strains** | **Specific growth rate (h^-1^)** | **Max OD** |
| --- | --- | --- |
| *E. coli* MG1655 | 0.39 | 0.89 |
| *E. coli* JM109 | 0.38 | 0.80 |
| *E. coli* BL21 (DE3) | 0.34 | 0.50 |
| *E. coli* HB101 | 0.37 | 0.78 |

**Table S9** Parameters of the pseudo-second-order swelling kinetic model fitting curves of the PAM-cell lysates.

| Material | We, cal | We, exp | *Ks* | *R^2^* |
| --- | --- | --- | --- | --- |
| PAM-cell lysates | 6.02 | 5.93 | 0.0163 | 0.996 |
